# Phosphine-Catalyzed Domino Regio- and Stereo-Selective Hexamerization of 2-(Bromomethyl)acrylates to 1,2-Bis(cyclohexenyl)ethenyl Derivatives

**DOI:** 10.1021/acs.orglett.3c02836

**Published:** 2023-09-29

**Authors:** Marta Papis, Raffaella Bucci, Alessandro Contini, Maria Luisa Gelmi, Leonardo Lo Presti, Giovanni Poli, Gianluigi Broggini, Camilla Loro

**Affiliations:** † Dipartimento di Scienza e Alta Tecnologia, Università degli Studi dell’Insubria, Via Valleggio 9, 22100, Como, Italy; ‡ Dipartimento di Scienze Farmaceutiche, DISFARM Università degli Studi di Milano, Via Venezian 21, 20133, Milano, Italy; § Dipartimento di Chimica, Università degli Studi di Milano, via Golgi 19, 20133 Milano, Italy; ∥ Sorbonne Université, Faculté des Sciences et Ingénierie, CNRS, Institut Parisien de Chimie Moléculaire, IPCM, 4 place Jussieu, 75005 Paris, France

## Abstract

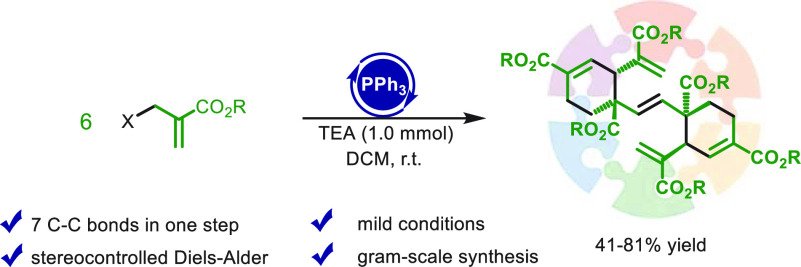

A phosphine-catalyzed domino assembly of six units of 2-bromomethyl acrylates afforded polyalkenyl adducts containing two cyclohexenyl rings. This reaction occurs under mild conditions providing the final product by formation of seven carbon–carbon bonds and four stereocenters. Experimental and computational studies support an initial dimerization of the substrate, which in turn trimerizes involving two totally regio- and stereocontrolled Diels–Alder cycloadditions. The yield of the hexamerization of the 2-bromomethyl acrylates depends on the size of the ester function. The protocol has also proved to be practicable on a gram scale.

Over the past two decades, nucleophilic phosphine catalysis has emerged as a powerful tool in organic synthesis.^1^ Specifically, the initial addition of a tertiary phosphine to an electrophilic π system generates a zwitterionic species that can in turn evolve in different ways, often in cascade processes.^2−4^ In this context, Morita–Baylis–Hillmann (MBH) adducts are very interesting electrophilic partners. Lu and co-workers reported the PPh_3_-catalyzed annulation between 2-halomethyl acrylates and *N*-phenyl-maleimide^5^ or tropone, to afford [3 + 3] or [3 + 6] cycloadducts, respectively.^6^ More recently, by using PCy_3_-catalyst, Huang described a [3 + 3] annulation between MBH carbonates and 4-amino-cyclohexandienones (Scheme 1, eq 1)^7^ as well as the sequential [2 + 4]/[2 + 3] annulation between MBH carbonates and 7-alkenyl-indoles (Scheme 1, eq 2).^8^ Finally, Guo discovered a Ph_2_PCy catalyzed annulation between diazenes and MBH carbonates (Scheme 1, eq 3).^9^

**Scheme 1:**
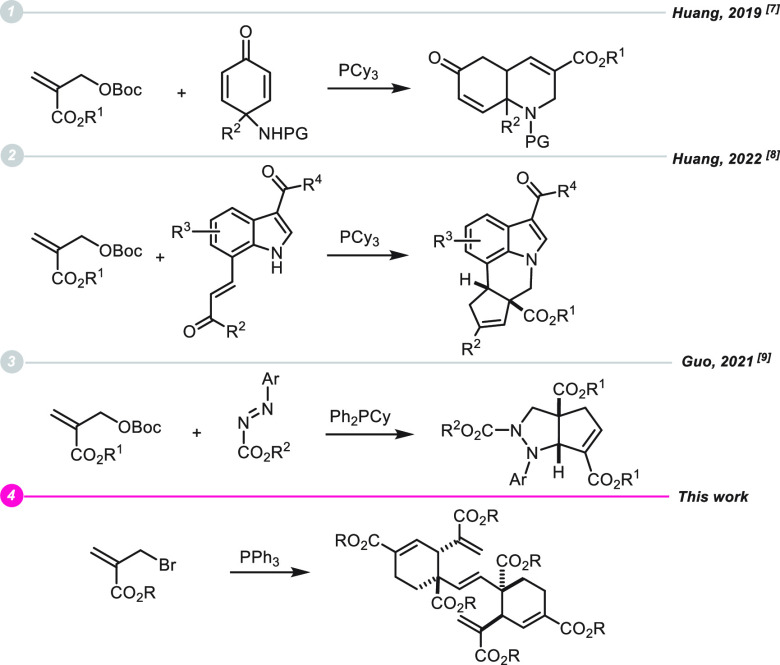
Selected Phosphine-Mediated Reactions Involving MBH Adducts or Derivatives

As part of our ongoing studies on the development of new domino processes,^10^ we report on a reaction that generates bicyclic structures through the assembly of six 2-(bromomethyl)acrylate units (Scheme 1, eq 4).

The treatment of methyl 2-(bromomethyl)acrylate (**1a**) with PPh_3_ (40 mol %) and triethylamine (1.0 mmol) for 24 h at room temperature afforded the bicyclic structure **2** in 63% yield, as confirmed by a single-crystal X-ray diffraction analysis (Scheme 2).

**Scheme 2:**
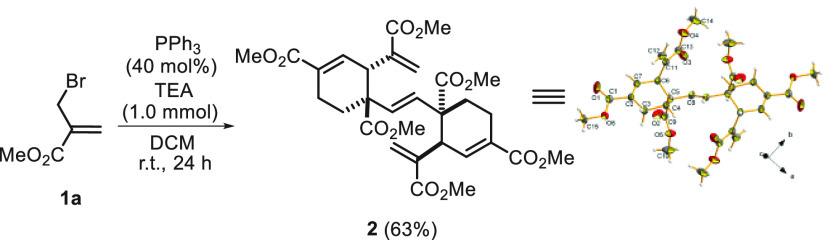
PPh_3_-Catalyzed Hexamerization of Methyl 2-(Bromomethyl)acrylate^,^^,^ Reaction conditions: methyl acrylate (1.0 mmol), PPh_3_ (0.4 mmol), TEA (1.0 mmol), DCM (0.1 M), r.t., 24 h. Isolation yields. CCDC 2286203 is the Cambridge Structural Database entry for **2**.

Such a striking totally regio- and stereoselective hexamerization involving the generation of seven C–C bonds and the control of four stereocenters prompted us to further investigate this reactivity. By extending the reaction time to 72 h, the yield was increased to 81% (Table 1, entry 1). By increasing the reaction temperature to 40 °C for 7 h or using 1.0 mmol of PPh_3_ for 24 h did not improve the yield (Table 1, entries 2, 3). Conversely, the use of a catalytic amount of PPh_3_ (10 mol %) gave only traces of the pentaenic product and a complex mixture of degradation products (Table 1, entry 4). The use of DIPEA, Na_2_CO_3_, or K_2_CO_3_ as bases, instead of TEA, led to unsatisfactory results (Table 1, entries 5–7). Replacing Ph_3_P with Cy_3_P, *n*Bu_3_P, or JohnPhos furnished only tarry products (Table 1, entries 8–10). On the other hand, the use of tri-2-furylphosphine led to **2** in 69% yield (Table 1, entry 11). The use of (±)-BINAP gave only traces of **2** at r.t., while heating the mixture at 40 °C allowed only a moderate yield improvement (Table 1, entries 12 and 13).

**Table 1:** Phosphine-Catalyzed Hexamerization of Methyl 2-(Bromomethyl)acrylate to **2**

entry^a^	PR_3_	base	temp (°C)	**2** (%)^b^
**1**^c^	**PPh**_**3**_	**TEA**	r.t.	**81**
2^d^	PPh_3_	TEA	40	77
3^e^	PPh_3_	TEA	r.t.	71
4^f^	PPh_3_	TEA	r.t.	traces
5	PPh_3_	DIPEA	r.t.	56
6	PPh_3_	Na_2_CO_3_	r.t.	degrad.
7	PPh_3_	K_2_CO_3_	r.t.	degrad.
8	PCy_3_	TEA	r.t.	degrad.
9	PBu^*n*^_3_	TEA	r.t.	degrad.
10	Johnphos	TEA	r.t.	degrad.
11	(2-Furyl)_3_P	TEA	r.t.	69
12	BINAP	TEA	r.t.	traces
13	BINAP	TEA	40	26

a Reaction conditions: **1a** (1.0 mmol), phosphine (40 mol %), base (1.0 mmol), DCM (0.1 M), 24 h.

b Isolation yields.

c Reaction time: 72 h.

d Reaction time: 7 h.

e PPh_3_ (1.0 mmol).

f PPh_3_ (10 mol %).

A set of additional experiments completed our initial study (Scheme 3). The hexamerization process also took place from methyl 2-chloromethyl acrylate **1b**, providing **2** in 78% yield. Conversely, the corresponding MBH acetate or carbonate was not reactive. Finally, repetition of the hexamerization of **1a** on a 3.0 mmol scale gave **2** in 76% yield after a 120 h reaction. Different MBH esters were next tested to check the scope of this new phosphine-catalyzed cascade reaction. Accordingly, ethyl, benzyl, *n*-butyl, and *tert*-butyl 2-(bromomethyl)acrylates (**1e**–**h**) smoothly afforded the corresponding pentaenic bicyclic structures **3**–**6** in variable yields depending on the steric hindrance of the ester. Conversely, 2-(bromomethyl)acrylic acid, 2-(bromomethyl)acryl *N,N*-dimethylamide, and the simple allyl bromide failed in the phosphine-catalyzed assembly.

**Scheme 3:**
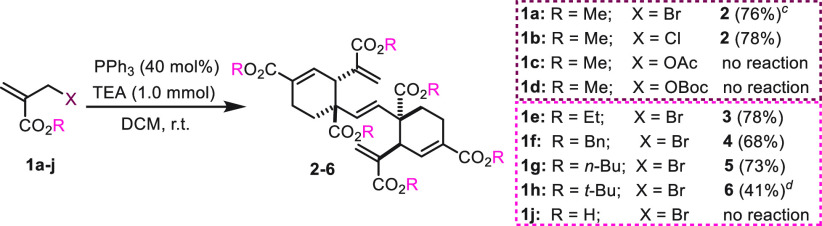
PPh_3_-Catalyzed Hexamerization of Differently SubstitutedAcrylates^,^ Reaction conditions: acrylates **1b**-**j** (1.0 mmol), PPh_3_ (0.4 mmol), TEA (1.0 mmol), DCM (0.1 M), r.t., 72 h. Isolation yields. Gram scale reaction: **1a** (3.0 mmol), PPh_3_ (1.2 mmol), TEA (3.0 mmol), DCM (0.1 M), r.t., 5 days. 5 days.

A possible reaction mechanism is proposed in Scheme 4 for compound **2**. Conjugate addition of triphenylphosphine to 2-(bromomethyl)acrylate followed by bromide elimination generates phosphonium bromide **II** via **I**, which, in the presence of triethylamine, gives the corresponding ylide **III**. A second conjugate addition/elimination sequence takes place between **III** and a new unit of acrylate to generate adduct **IV**. Subsequent deprotonation of **IV** by triethylamine triggers triphenylphosphine elimination with generation of conjugated triene **VI** via **V**. However, as the most acidic H atom in **IV** is on the carbon atom directly linked to the phosphorus atom, the generation of **V** may pass through the reversible formation of an unproductive ylide (not shown), or ylide formation is followed by a 1,2 proton shift.

**Scheme 4:**
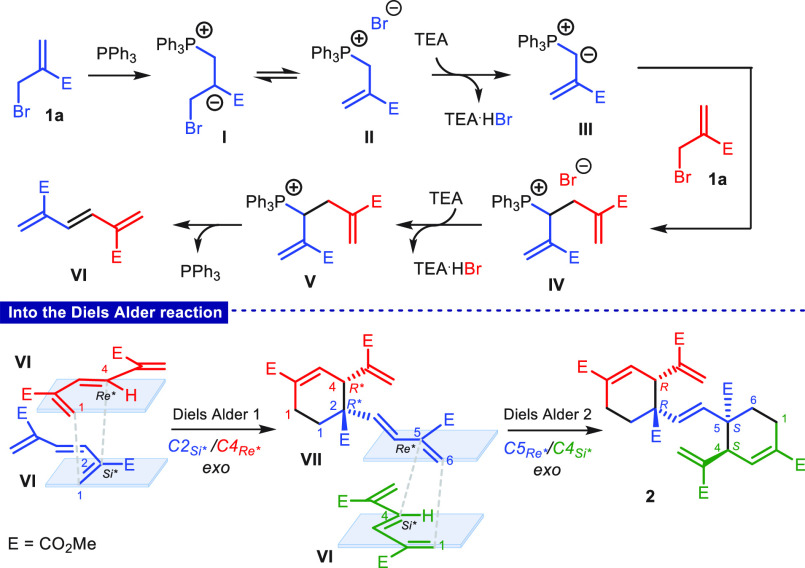
Proposed Mechanism for the Conversion of 2-(Bromomethyl)acrylate **1a** into Dicyclohexenyl Product **2**

From this point, the generation of pentaenic product **2** appears to derive from two consecutive Diels–Alder (DA) cycloadditions involving three units of key triene **VI**. In particular, while two units of **VI** act as dienes, the third one plays the role of a double dienophile. The formation of **2** as single regio- and stereoisomer of *C*_*i*_ point group symmetry implies that an *exo*-control (C2 dienophile/C4 diene Si*/Re*) is at work during the first cycloaddition to give intermediate **VII**, while an opposite *exo*-control (C5 dienophile/C4 diene Re*/Si*) takes place in the second cycloaddition. This means that besides the regioselectivity, a total diastereoselectivity is at work in both of the cycloadditions.

Since the proposed mechanism is based on the involvement of triene **VI**, it was essential to prove the formation of this key intermediate. Despite several trials, detection of **VI** in crude reaction mixtures, even after short reaction times, was fruitless. Hence, indirect detection of **VI** was planned. We chose a nitrile oxide as a trapping agent, as this 1,3-dipole is known to regioselectively react with electron-poor dipolarophiles.^11^ After a 15 min exposure of 2-(bromomethyl)acrylate to triphenylphosphine and triethylamine, the addition of chloroxime **7** and triethylamine (to generate benzonitrile oxide) afforded the centrosymmetric bis-isoxazoline **8** as the sole product (Scheme 5). Again, the double cycloaddition was totally regio- and stereoselective, as confirmed by X-ray diffraction analysis of a single crystal of **8**.

**Scheme 5:**
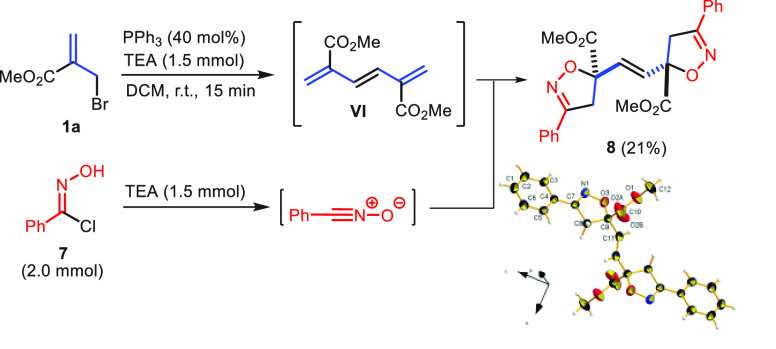
Capture of Triene Intermediate by 1,3-Dipolar Cycloaddition^,^^,^ Reaction conditions: step 1: **1a** (1.0 mmol), PPh_3_ (0.4 mmol), TEA (1.5 mmol), DCM (0.1 M), r.t., 15 min.; step 2: **7** (2.0 mmol), TEA (1.5 mmol), r.t., 24 h. Isolation yields. CCDC 2286201 is the Cambridge Structural Database entry for **8**.

Given the presence of five ethylenic bonds in **2**, we considered its functionalization via 1,3-dipolar cycloaddition to increase the molecular complexity. Accordingly, treatment of **2** with benzonitrile oxide (*in situ* generated from **7** and TEA) afforded the tetracyclic centrosymmetric bis-isoxazole **9** in 56% yield as the sole product, whose structure was confirmed by X-ray diffraction analysis (Scheme 6, *path B*). So, once again, the reaction was totally regio- and stereoselective. Compound **9** could also be obtained in good yield (48%) in a *one-pot* process by generating *in situ* benzonitrile oxide in the presence of **2**, which was in turn *in situ* generated from **1a**, providing on the whole a dimerization/double DA cycloaddition/double 1,3-dipolar cycloaddition process (Scheme 6, *path A*).

**Scheme 6:**
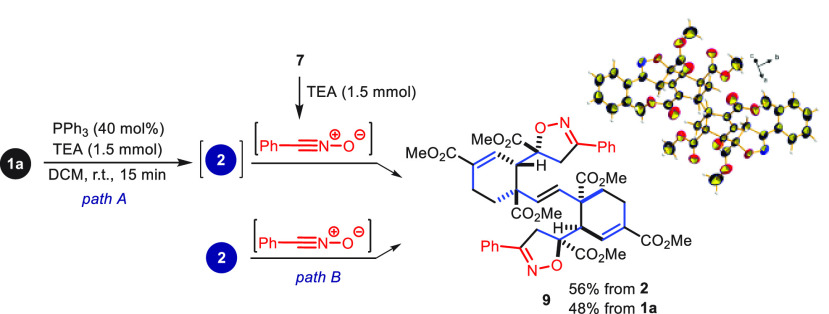
Dimerization/Diels–Alder/1,3-Dipolar Cycloadditions from **1a**^,^^,^ Reaction conditions: *path A*: **1a** (1.0 mmol), PPh_3_ (0.4 mmol), DCM (0.1 M), r.t., 72 h; then: **7** (2.0 mmol), TEA (1.5 mmol), r.t., 24 h; *path B*: **2** (1.0 mmol), **7** (2.0 mmol), TEA (1.5 mmol), DCM (0.1 M), r.t., 24 h. Isolation yields. CCDC 2286202 is the Cambridge Structural Database entry for **9**.

To understand the reason for such a total stereoselectivity, DFT calculations were performed.^12,13^ The lowest energy geometry (Table S1, Supporting Information (SI)) for each ground state and transition state (TS) was used for evaluating the enthalpy (Figure 1A and B) and free energy (Figure S1A and B; SI) paths of the first and second cycloaddition. As to the first cycloaddition, we modeled the reaction between two molecules of monomer **VI** to provide intermediate **VII** in both the 3*R*,4*R* (**VII**-*RR*) and 3*R*,4*S* (**VII**-*RS*) stereochemistries. We found that the path leading to the former stereoisomer was kinetically favored over the second, with activation barriers (*ΔH*^*‡*^) = 9.2 and 15.6 kcal/mol, respectively, from the activated complex (AC-**VII** vs TS-**VII**). No relevant difference was found in reaction enthalpy (*ΔH*), suggesting that **VII**-*RR* and **VII**-*RS* are thermodynamically equivalent (Figure 1A). As to the second cycloaddition, we investigated the reaction between the kinetically favored **VII**-*RR* and monomer **VI**, leading to the **2**-*RRSS* and **2**-*RRRR* diastereoisomers (Figure 1B). In this case, the former compound was favored over **2**-*RRRR* both kinetically (*ΔH*^*‡*^ = 9.3 and 16.9 kcal/mol, respectively; AC-**2** vs TS-**2**) and thermodynamically (*ΔH* = −33.0 and −23.3 kcal/mol, respectively; **2** vs AC-**2**).

**Figure 1:**
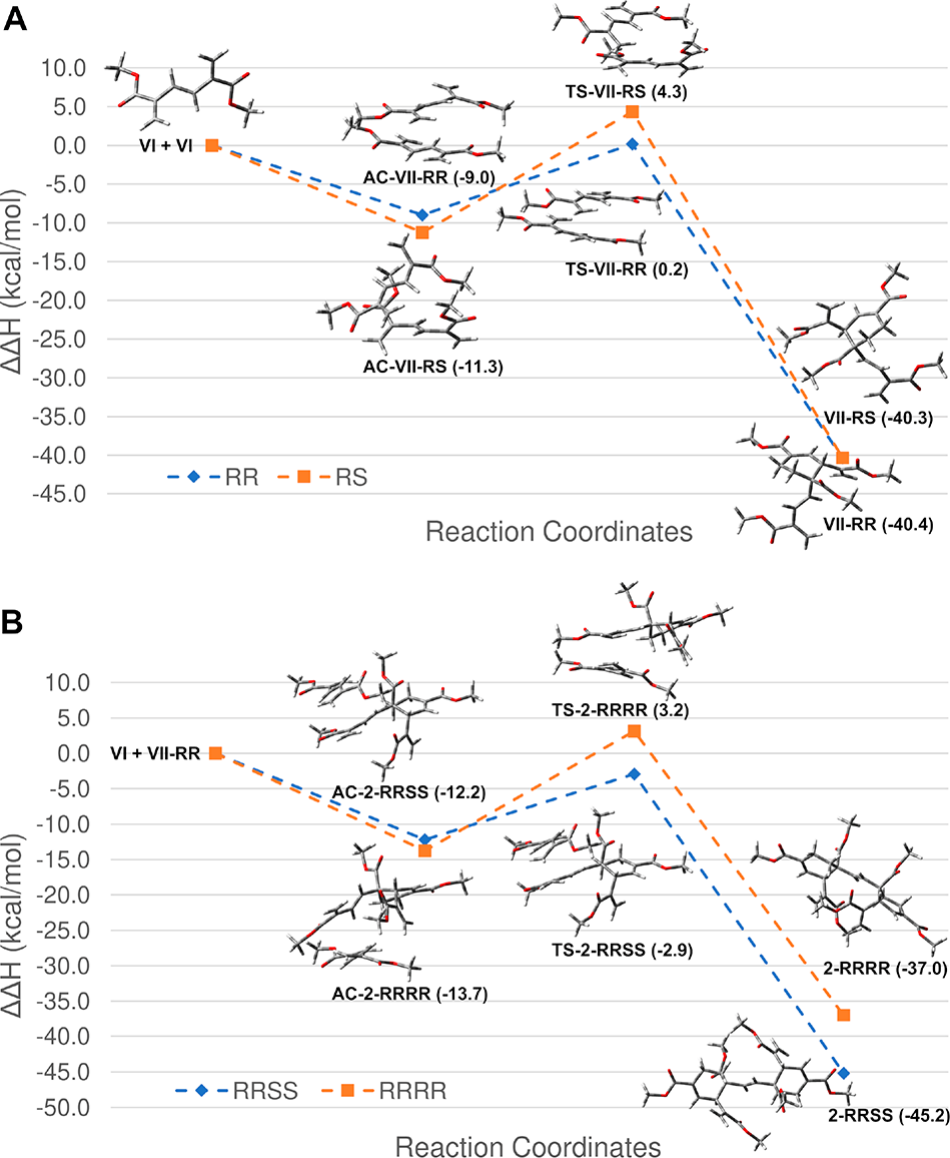
DFT and QTAIM analysis of the reaction mechanism leading to experimentally isolated and nonisolated stereoisomers. (A) Enthalpy path and stationary points for the dimerization of **VI**, leading to **VII**-*RS* and **VII**-*RR*. (B) Enthalpy path and stationary points for the addition of **VII**-*RR* to **VI**, leading to **2**-*RRRR* (not isolated) and **2**-*RRSS* (isolated) stereoisomers. *ΔH* values relative to the isolated reactants are reported in parentheses in kcal/mol.

We analyzed the difference between TS-**2**-*RRSS* and TS-**2**-*RRRR* by performing a topological analysis of the electron density using the Bader’s Quantum Theory of Atoms in Molecules (QTAIM).^14,15^ In QTAIM, both covalent and noncovalent interactions are defined by a bond path (BP) and by a bond critical point (BCP). The value of electron density ρ(r) at the BCP is a measure of the strength of the interaction. Results are summarized in Figure S2A, B (SI) and Table S2 (SI), where BPs connecting noncovalently bound oxygen and hydrogens (HB) are reported with the corresponding BCPs. From the molecular graphs, it can be observed that four HB BCPs (BCP1–4) are found for TS-**2**-*RRRR* (Figure S2A, SI), three of which belong to intermolecular BPs connecting the two reactants. Conversely, eight HB BCPs were found in TS-**2**-*RRSS*, six of which were intermolecular. Additionally, the total electron density ρ(r) of HB BCPs is 0.066659 and 0.047609 au for TS-**2**-*RRSS* and TS-**2**-*RRRR*, respectively. This indicates stronger, as well as more numerically abundant, interactions among the reactants in the former TS, justifying the selectivity observed both theoretically and experimentally.

In conclusion, we have disclosed a highly effective phosphine-catalyzed procedure that allows assembly, in a totally regio- and stereoselective way, of six molecules of 2-(bromomethyl)acrylates through the formation of seven carbon–carbon bonds and four stereocenters. The resulting sole product is a centrosymmetric pentaene containing two cyclohexenyl units derived from a dimerization/double DA cycloaddition sequence. A key intermediate of this domino sequence is the 2,5-dicarbomethoxy-1,3,5-triene **VI**, whose formation was evidenced by its trapping through a 1,3-dipolar cycloaddition with benzonitrile oxide. Furthermore, adduct **2** was also found to undergo a double and totally selective 1,3-dipolar cycloaddition with benzonitrile oxide, generating a tetracyclic bis-isoxazole adduct as the sole product. DFT computations of the two DA steps supported the proposed mechanism. Computed *ΔH*^*‡*^ are consistent with a reaction occurring at room temperature as well as with the observed selectivity. Future studies will be directed toward expanding the scope of the reaction between **1** and other 1,3-dipoles to achieve new structures and higher complexity.

## Data Availability

The data underlying this study are available in the published article and its Supporting Information.

## Supporting Information Available

Experimental procedures, compound characterization data including copies of ^1^H and ^13^C NMR spectra, computational details, and crystallographic data for compounds **2**, **8**, and **9**. (PDF)
