# Supplementary material for: Phosphine-Catalyzed Domino Regio- and Stereo-Selective Hexamerization of 2-(Bromomethyl)acrylates to 1,2-Bis(cyclohexenyl)ethenyl Derivatives
Source: Org Lett. 2023 Sep 29;25(40):7380–4. doi: 10.1021/acs.orglett.3c02836 (PMC10580324; doi:10.1021/acs.orglett.3c02836)
Supplement: Supplementary file 1 — ol3c02836_si_001.pdf [file ol3c02836_si_001.pdf]

Phosphine-Catalyzed Domino Regio- and Stereo-Selective Hexamerization of 2-(Bromomethyl)acrylates to 1,2-Bis(cyclohexenyl)ethenyl Derivatives

Marta Papis,[a] Raffaella Bucci,[b] Alessandro Contini,[b] Maria Luisa Gelmi,[b] Leonardo Lo Presti,[c] Giovanni Poli,[d] Gianluigi Broggini[a] and Camilla Loro*[a]

[a] Dipartimento di Scienza e Alta Tecnologia, Università degli Studi dell'Insubria, Via Valleggio 9, 22100, Como, Italy

[b] Dipartimento di Scienze Farmaceutiche, DISFARM Università degli Studi di Milano, Via Venezian 21, 20133, Milano, Italy

[c] Dipartimento di Chimica, Università degli Studi di Milano, via Golgi 19, 20133 Milano, Italy

[d] Sorbonne Université, Faculté des Sciences et Ingénierie, CNRS, Institut Parisien de Chimie Moléculaire, IPCM, 4 place Jussieu, 75005 Paris, France

Table of contents

General information S-2

Synthesis of starting materials S-3

General procedure for hexamerization of 2-(bromomethyl)acrylates S-4

Gram-scale synthesis of compound **2** S-6

Cycloaddition procedures S-6

Procedure for the synthesis of *N*-hydroxybenzimidoyl chloride **7** S-6

Procedure for the synthesis of compound **8** S-6

Procedure for the synthesis of compound **9** S-7

Computational methods and references S-8

Table S1. Total energies and thermochemical corrections S-8

Figure S1. DFT free energy path for first and second cycloaddition S-9

Figure S2. QTAIM analysis for **2**-*RRSS* and **2**-*RRRR* S-10

Table S2. Hydrogen bonds BCPs and corresponding ρ(r) found by QTAIM analysis S-10

Figure S3. DFT geometry of **2**-*RRSS* as found in the global minimum and X-ray S-11

Figure S4. Geometries of TSs for the first cycloaddition S-12

Figure S5. Geometries of TSs for the second cycloaddition S-12

Figure S6. Intrinsic reaction coordinate analyses for the first cycloaddition S-13

Figure S7. Intrinsic reaction coordinate analyses for the second cycloaddition S-13

Cartesian Coordinates in Gaussian Input file format S-14

Crystallographic data for compounds **2**, **8** and **9** S-21

^{1}H and ^{13}C NMR Spectra S-27

General Information

All available chemicals and solvents were purchased from commercial sources and were used without any further purification. Thin layer chromatography (TLC) was performed using 0.25 mm silica gel precoated plates Si 60-F254 (Merck, Darmstadt, Germany) visualized by UV-254 light and CAM staining. Purification by flash column chromatography (FCC) was conducted by using silica gel Si 60, 230-400 mesh, 0.040-0.063 mm (Merck). Melting points were determined on a Stuart Scientific SMP3 and are corrected. ^{1}H and ^{13}C NMR spectra were recorded on a Bruker Avance 400 (400 and 101 MHz, respectively); chemical shifts are indicated in parts per million downfield from $SiMe_4$, using the residual proton ($CHCl_3$ = 7.27 ppm) and carbon ($CDCl_3$ = 77.0 ppm) solvent resonances as internal reference. Coupling constants values *J* are given in Hz. FT-IR spectra were recorded on a Tensor 27 (ATR Diamond) Bruker infrared spectrophotometer and are reported in frequency of absorption (cm^{-1}). Elemental analyses were executed on Perkin-Elmer CHN Analyzer Series II 2400.

Synthesis of starting materials

General procedure for the synthesis of 2-(hydroxymethyl)acrylates

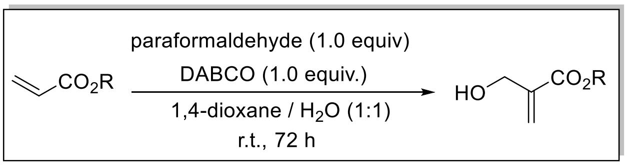

In a round bottom flask, paraformaldehyde (1.0 equiv., 1.0 mmol, 30.0 mg), DABCO (1.0 equiv., 1.0 mmol, 112.2 mg) were added to the solution of the appropriate acrylate (3.0 equiv., 3.0 mmol) in 1,4-dioxane-H_2O (1:1, 0.1 M). The resulted solution was magnetically stirred at room temperature for 72 hours. The reaction mixture was washed with brine (3 x 5 mL) and the organic layer was extracted with AcOEt (2 x 5 mL), dried over $MgSO_4$ and filtered. The solvent was evaporated under reduced pressure. The residue was purified by FCC. Starting from appropriate acrylate, yield and physical, spectroscopic and analytical data of compounds are as follows.

Benzyl-2-(hydroxymethyl)acrylate

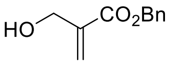

Benzyl acrylate (162.2 mg); FCC–AcOEt/hexane (3:7). Benzyl-2-(hydroxymethyl)acrylate (155.6 mg, 81%); colorless oil. ^{1}H NMR ($CDCl_3$, 400 MHz) δ 7.39-7.33 (m, 5H), 6.33 (s, 1H), 5.87 (d, 1H, *J* = 0.9 Hz), 5.24 (s, 2H), 4.36 (s, 2H), 2.12 (s, 1H). The characterization of benzyl-2-(hydroxymethyl)acrylate was consistent with that reported in literature.[1]

Butyl-2-(hydroxymethyl)acrylate

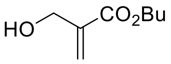

Butyl acrylate (128.2 mg); FCC–AcOEt/PE (3:7). Butyl-2-(hydroxymethyl)acrylate (118.6 mg, 75%); colorless oil. ^{1}H NMR ($CDCl_3$, 400 MHz) δ 6.24 (s, 1H), 5.82 (s, 1H), 4.31 (s, 2H), 4.19 (t, 2H, *J* = 6.6 Hz), 2.49 (s, 1H), 1.69-1.62 (m, 2H), 1.45-1.35 (m, 2H), 0.94 (t, 3H, *J* = 7.8 Hz). The characterization of butyl-2-(hydroxymethyl)acrylate was consistent with that reported in literature.[2]

t-Butyl-2-(hydroxymethyl)acrylate

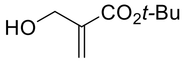

t-Butyl acrylate (128.2mg); FCC–AcOEt/hexane (3:7). *t*-Butil-2-(hydroxymethyl)acrylate (112.2 mg, 71%); colorless oil. ^{1}H NMR ($CDCl_3$, 400 MHz) δ 6.06 (s, 1H), 5.69 (s, 1H), 4.18 (d, 2H, *J* = 4.2 Hz), 3.18 (t, 1H, *J* = 5.2 Hz), 1.41 (s, 9H). The characterization of *t*-butyl-2-(hydroxymethyl)acrylate was consistent with that reported in literature.[2]

General procedure for the synthesis of 2-(bromomethyl)acrylates 1f-h

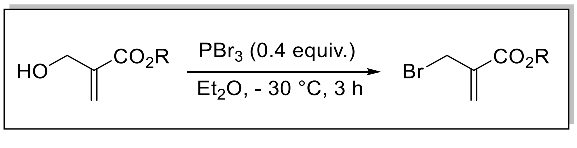

The appropriate 2-(hydroxymethyl)acrylate (1.0 equiv., 1.0 mmol) was dissolved in anhydrous diethylether (0.3 M) and the reaction mixture cooled to -30 °C. Then PBr_3 (1.1 equiv., 1.1 mmol, 297.8 mg) was added and then the mixture was allowed to warm up to 0 °C within 3 hours. After cooling to -10 °C the reaction was quenched by the addition of water (2 mL), warmed to

[1] Ischenko, A. Y.; Yanik, S.; Rusanov, E. B.; Komarov, I. V.; Kirby, A. An Expedient and Practical Approach to Functionalized 3-Aza-, 3-Oxa, and 3-Thiabicyclo [3.3.1]nonane Systems. *Synthesis* **2015**, *47*, 367-376.

[2] Peng, C. ; Joy, A. Baylis-Hillman Reaction as a Versatile Platform for the Synthesis of Diverse Functionalized Polymers by Chain and Step Polymerization. *Macromolecules* **2014**, *47*, 1258-1268.

room temperature and dried over $MgSO_4$. The solvent was evaporated under reduced pressure. Starting from appropriate 2-(hydroxymethyl)acrylate, yield and physical, spectroscopic and analytical data of compounds **1f-h** are as follows.

Benzyl-2-(bromomethyl)acrylate (1f)

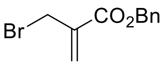

1f

Benzyl 2-(hydroxymethyl)acrylate (192.1 mg). **1f** (236.2 mg, 93%); colorless oil. ^1H NMR ($CDCl_3$, 400 MHz) δ 7.42-7.33 (m, 5H), 6.39 (s, 1H), 5.99 (s, 1H), 5.27 (s, 2H), 4.21 (s, 2H). The characterization of product **1f** was consistent with that reported in literature.[1]

Butyl-2-(bromomethyl)acrylate (1g)

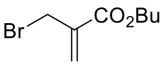

1g

Butyl 2-(hydroxymethyl)acrylate (158.1 mg). **1g** (211.2 mg, 96%); colorless oil. ^1H NMR ($CDCl_3$, 400 MHz) δ 6.31 (s, 1H), 5.92 (s, 1H), 4.20 (t, 2H, J = 6.6 Hz), 4.17 (s, 2H), 1.69-1.63 (m, 2H), 1.44-1.37 (m, 2H), 0.94 (t, 3H, J = 7.3 Hz). The characterization of product **1g** was consistent with that reported in literature.[3]

***t*-Butyl-2-(bromomethyl)acrylate (1h)**

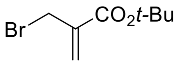

1h

t-Butyl 2-(hydroxymethyl)acrylate (158.1 mg).**1h** (195.8 mg, 89%); colorless oil. ^1H NMR ($CDCl_3$, 400 MHz) δ 6.24 (s, 1H), 5.87 (s, 1H), 4.16 (s, 2H), 1.53 (s, 9H). The characterization of product 1d was consistent with that reported in literature.[3]

General procedure for the hexamerization of 2-(bromomethyl)acrylates: synthesis of compounds 2-6

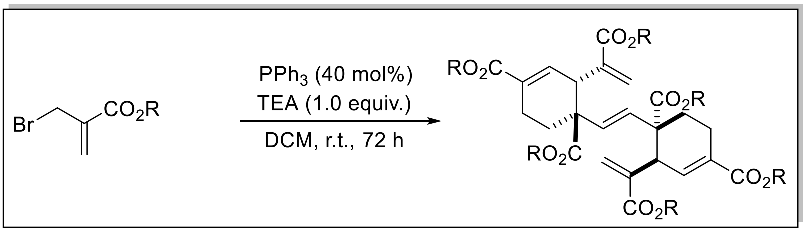

In a round bottom flask, PPh_3 (0.4 equiv., 0.4 mmol, 104.9 mg), triethylamine (1.0 equiv., 1.0 mmol, 101.2 mg) were added to the solution of the appropriate 2-(bromomethyl)acrylate (1.0 equiv., 1.0 mmol) in DCM dry (0.1 M). The resulted solution was magnetically stirred at room temperature for 72 hours. The reaction mixture was washed with brine (3 x 5 mL) and the organic layer was extracted with DCM (2 x 5 mL), dried over MgSO4 and filtered. The solvent was evaporated under reduced pressure. The residue was purified by FCC. Starting from aromatic source, appropriate sulfonamide, yield and physical, spectroscopic and analytical data of compounds **2-6** are as follows.

1,2-Bis[2-(1-(methoxycarbonyl)eth-1-en-1yl)-1,4-di(methoxycarbonyl)cyclohex-3-en-1-yl]ethene (2)

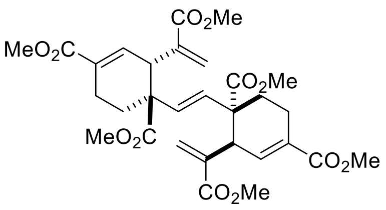

2

Methyl 2-(bromomethyl)acrylate (179.0 mg); FCC–AcOEt/hexane (3:2). **2** (79.4 mg, 81%); white solid; M.p.: 192-193 °C. ^1H NMR ($CDCl_3$, 400 MHz) δ 6.75 (d, 1H, J = 4.7 Hz), 6.38 (s, 1H), 5.45 (s, 1H), 5.36 (s, 1H), 4.51 (d, 1H, J = 3.3 Hz), 3.78 (s, 3H), 3.72 (s, 3H), 3.63 (s, 3H), 2.46 (dd, 1H, J = 18.7, 5.4 Hz), 2.29-2.22 (m, 1H), 1.97 (dd, 1H, J = 13.4, 6.1 Hz), 1.59-1.51 (m, 1H); ^{13}C NMR ($CDCl_3$, 101 MHz) δ 173.5, 167.2, 167.0, 138.9, 138.5, 130.6, 130.5, 129.9, 52.2 ($2CH_3$), 51.7, 50.5, 40.7, 21.9, 21.6; IR ν_{max} 1732, 1709, 1652 cm^{-1}. Anal. Calcd. For $C_{30}H_{36}O_{12}$: C, 61.22; H, 6.17. Found: C, 61.29; H, 6.09.

[3] Jiang, B. ; Meng, F.-F. ; Liang, Q.-L. ; Xu, Y.-H. ; Loh T.-P. Palladium-Catalyzed Direct Intramolecular C-N Bond Formation : Access to Multisubstituted Dihydropyrroles. *Org. Lett.* **2017**, *19,* 914-917.

1,2-Bis[2-(1-(ethoxycarbonyl)eth-1-en-1yl)-1,4-di(ethoxycarbonyl)cyclohex-3-en-1-yl]ethene (3)

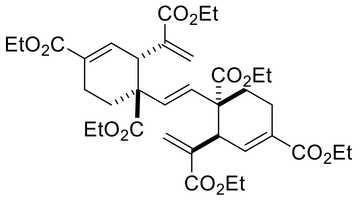

3

Ethyl 2-(bromomethyl)acrylate (193.0 mg); FCC–AcOEt/hexane (3:2). **3** (87.4 mg, 78%); white solid; M.p.: 159-164 °C. ^{1}H NMR ($CDCl_3$, 400 MHz) δ 6.75 (d, 1H, *J* = 5.2 Hz), 6.38 (s, 1H), 5.50 (s, 1H), 5.36 (s, 1H), 4.52 (d, 1H, *J* = 3.2 Hz), 4.33-4.23 (m, 1H), 4.19-4.13 (m, 4H), 4.11-4.03 (m, 1H), 2.44 (dd, 1H, *J* = 19.1, 6.2 Hz), 2.38-2.29 (m, 1H), 2.00 (dd, 1H, *J* = 13.7, 5.9 Hz), 1.55-1.50 (m, 1H), 1.34 (t, 3H, *J* = 7.1 Hz), 1.28 (t, 3H, *J* = 7.1 Hz), 1.20 (t, 3H, *J* = 7.1 Hz); ^{13}C NMR ($CDCl_3$, 101 MHz) δ 172.9, 166.8, 166.6, 139.3, 138.4, 130.8, 130.6, 129.6, 61.3, 61.1, 60.5, 50.2, 40.6, 21.9, 21.8, 14.2, 14.1, 14.0; IR ν_{max} 1722, 1702, 1651 cm^{-1}. Anal. Calcd. For $C_{36}H_{48}O_{12}$: C, 64.27; H, 7.19. Found: C, 64.41; H, 7.01.

1,2-Bis[2-(1-(benzyloxycarbonyl)eth-1-en-1yl)-1,4-di(benzyloxycarbonyl)cyclohex-3-en-1-yl]ethene (4)

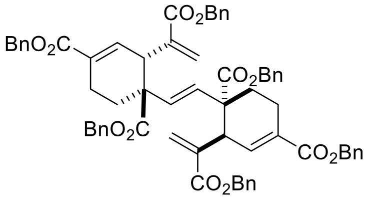

4

Benzyl 2-(bromomethyl)acrylate (253.9 mg); FCC–AcOEt/hexane (3:2). **4** (118.4 mg, 68%); yellow oil. ^{1}H NMR ($CDCl_3$, 400 MHz) δ 7.28-7.09 (m, 15H), 6.70 (d, 1H, *J* = 3.6 Hz), 5.41 (s, 1H), 5.18 (s, 2H), 5.08 (q, 2H, *J* = 6.5 Hz), 4.93 (s, 3H), 4.48 (d, 1H, *J* = 2.6 Hz), 2.32-2.18 (m, 2H), 1.84 (dd, 1H, *J* = 13.6, 5.3 Hz), 1.37-1.29 (m, 2H); ^{13}C NMR ($CDCl_3$, 101 MHz) δ 172.5, 166.4, 166.3, 139.0, 138.5, 136.0, 135.96, 135.93, 130.9, 130.6, 130.5, 128.6, 128.5, 128.4, 128.2, 128.15, 128.12, 128.09, 128.0, 127.9, 66.9, 66.6, 66.3, 50.4, 40.7, 21.9, 21.7; IR ν_{max} 1706, 1652 cm^{-1}. Anal. Calcd. For $C_{66}H_{60}O_{12}$: C, 75.84; H, 5.79. Found: C, 75.75; H, 5.94.

1,2-Bis[2-(1-(butyloxycarbonyl)eth-1-en-1yl)-1,4-di(butyloxycarbonyl)cyclohex-3-en-1-yl]ethene (5)

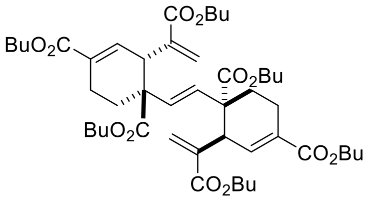

5

Butyl 2-(bromomethyl)acrylate (220.0 mg); FCC–AcOEt/hexane (3:2). **5** (102.3 mg, 73%); yellow oil. ^{1}H NMR ($CDCl_3$, 400 MHz) δ 6.74 (d, 1H, *J* = 2.4 Hz), 6.37 (s, 1H), 5.50 (s, 1H), 5.35 (s, 1H), 4.52 (d, 1H, *J* = 2.9 Hz), 4.25-4.01 (m, 6H), 2.45-2.35 (m, 2H), 2.12-1.93 (m, 2H), 1.69-1.55 (m, 6H), 1.46-1.30 (m, 6H), 0.94-0.91 (m, 9H); ^{13}C NMR ($CDCl_3$, 101 MHz) δ 171.7, 165.8, 165.6, 139.3, 137.2, 129.9, 129.7, 128.6, 64.2, 63.9, 63.4, 49.3, 39.6, 29.6 (2CH_2), 29.5, 20.9, 20.8,18.3, 18.2, 18.1, 12.8, 12.7, 12.6; IR ν_{max} 1711, 1651 cm^{-1}. Anal. Calcd. For $C_{48}H_{72}O_{12}$: C, 68.55; H, 8.63. Found: C, 68.42; H, 8.78.

1,2-Bis[2-(1-(*t*-butyloxycarbonyl)eth-1-en-1yl)-1,4-di(*t*-butyloxycarbonyl)cyclohex-3-en-1-yl]ethene (6)

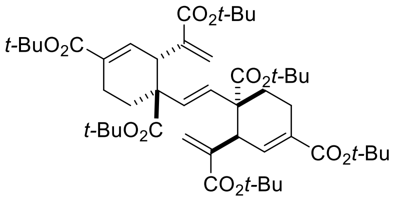

6

t-Butyl 2-(bromomethyl)acrylate (220.0 mg), reaction time: 5 days; FCC–AcOEt/hexane (3:2). **6** (57.4 mg, 41%); white solid; M.p.: 159-164 °C. ^{1}H NMR ($CDCl_3$, 400 MHz) δ 6.64 (d, 1H, *J* = 3.5 Hz), 6.28 (s, 1H), 5.55 (s, 1H), 5.32 (s, 1H), 4.46 (d, 1H, *J* = 2.6 Hz), 2.47-28 (m, 2H), 2.17 (dd, 1H, *J* = 13.4, 4.2 Hz), 1.87 (dd, 1H, *J* = 16.4, 6.5 Hz), 1.54 (s, 9H), 1.46 (s, 9H), 1.38 (s, 9H); ^{13}C NMR ($CDCl_3$, 101 MHz) δ 172.0, 166.4, 165.9, 141.1, 138.2, 131.14, 131.13, 128.9, 81.1, 80.8, 80.1, 50.7, 40.3, 28.1, 28.0, 27.9, 22.7, 21.7; IR ν_{max} 1703, 1652 cm^{-1}. Anal. Calcd. For $C_{48}H_{72}O_{12}$: C, 68.55; H, 8.63. Found: C, 68.70; H, 8.47.

Gram-scale synthesis of compound 2

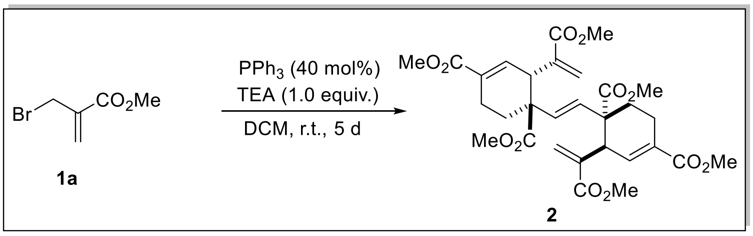

In a round bottom flask, PPh_3 (0.4 equiv., 2.0 mmol, 524,6 mg), triethylamine (1.0 equiv., 5.0 mmol, 506.0 mg) were added to the solution of the methyl 2-(bromomethyl)acrylate **1a** (1.0 equiv., 5.0 mmol, 895.1 mg) in DCM dry (0.1 M). The resulted solution was magnetically stirred at room temperature for 5 days. The reaction mixture was washed with brine (3 x 20 mL) and the organic layer was extracted with DCM (2 x 20 mL), dried over MgSO4 and filtered. The solvent was evaporated under reduced pressure. Compound **2** was obtained (372.5 mg, 76%) as a white solid after FCC– AcOET/hexane (3:2).

Cycloaddition procedures

Procedure for the synthesis of *N*-hydroxybenzimidoyl chloride 7

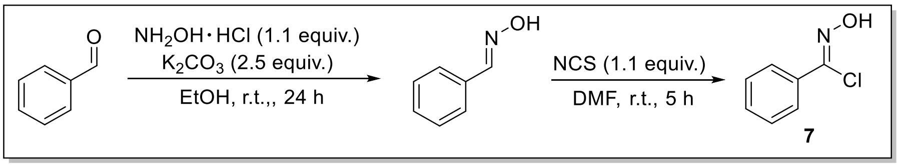

In a round bottom flask, K_2CO_3 (2.5 equiv., 2.5 mmol, 345.5 mg) and $NH_2OH \cdot HCl$ (1.1 equiv., 1.1 mmol, 76.4 mg) were added to the solution of the benzaldehyde (1.0 equiv., 1.0 mmol, 106.1 mg) in ethanol (2.0 M). The resulted solution was magnetically stirred at room temperature for 24 hours. The reaction mixture was washed with brine (3 x 5 mL) and the organic layer was extracted with AcOEt (2 x 5 mL), dried over MgSO4 and filtered. The solvent was evaporated under reduced pressure. Banzaldehyde oxime was afforded (130.5 mg, 93%) as white solid. ^1H NMR ($CDCl_3$, 400 MHz) δ 8.68 (s, 1H), 8.18 (s, 1H), 7.65-7.66 (m, 2H), 7.45-7.35 (m, 3H). The characterization of the benzaldehyde oxime was consistent with that reported in literature.[4]

In a round bottom flask, the benzaldehyde oxime (1.0 equiv., 1.0 mmol, 121.1 mg) was dissolved in dry DMF (1.2 M) and the reaction mixture was cooled to 0 °C. Then, the *N*-chloro succinimide (1.1 equiv., 1.1 mmol, 146.9 mg) was added. The resulted solution was magnetically stirred at room temperature for 4 hours. The reaction mixture was washed with brine (3 x 5 mL) and the organic layer was extracted with AcOEt (2 x 5 mL), dried over MgSO4 and filtered. The solvent was evaporated under reduced pressure. Compound **7** was obtained (125.6 mg, 81%) as pale yellow oil.^1H NMR ($CDCl_3$, 400 MHz) δ 9.15 (s, 1H), 7.85 (d, 2H, J = 7.0 Hz), 7.44-7.41 (m, 3H). The characterization of oxime was consistent with that reported in literature.[4]

Procedure for the synthesis of compound 8

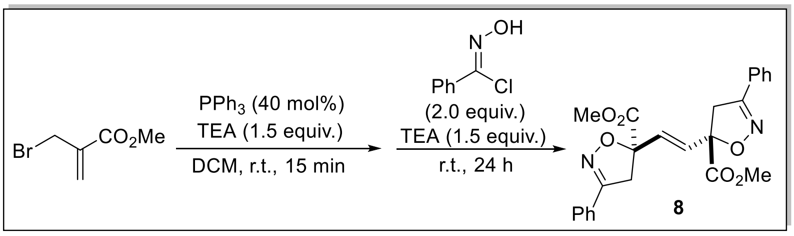

[4] Sanders, B. C.; Friscourt, F.; Ledin, P. A.; Mbua, N. E.; Arumugam, S.; Guo, J.; Boltje, T. J.; Popik, V. V.; Boons, G.-J. Metal-Free Sequential [3+2]-Dipolar Cycloadditions using Cyclooctynes and 1,3-Dipoles of Different Reactivity. *J. Am. Chem. Soc.* **2011**, *133*, 949-957.

In a round bottom flask after 15 minutes at room temperature, triethylamine (1.5 equiv., 1.5 mmol, 151.8 mg) and the chloro-oxime **7** (2.0 equiv., 2.0 mmol, 310.0 mg) were added to the solution of methyl 2-(bromomethyl)acrylate **1a** (1.0 equiv., 1.0 mmol, 179.0 mg), PPh_3 (0.4 equiv., 0.4 mmol, 104.9 mg) and triethylamine (1.5 equiv., 1.5 mmol, 151.8 mg) in DCM (0.1 M). The resulted solution was magnetically stirred at room temperature for 24 hours. The reaction mixture was washed with brine (3 x 5 mL) and the organic layer was extracted with DCM (2 x 5 mL), dried over MgSO4 and filtered. Compound **8** was obtained (45.6 mg, 21%) as white solid after FCC – AcOEt/PE (3:7); m.p.: 224-226 °C. ^{1}H NMR ($CDCl_3$, 400 MHz) δ 7.66 (d, 2H, *J* = 6.2 Hz), 7.43-7.42 (m, 3H), 6.37 (s, 1H), 3.99 (d, 1H, *J* = 17.0 Hz), 3.79 (s, 3H), 3.49 (d, 1H, *J* = 17.0 Hz); ^{13}C NMR ($CDCl_3$, 101 MHz) δ 170.7, 156.1, 130.6, 129.0, 128.8, 128.5, 126.9, 87.7, , 53.4, 45.0. IR ν_{max} 1707, 1653 cm^{-1}. Anal. Calcd. For $C_{24}H_{22}N_2O_6$: C, 66.35; H, 5.10; N, 6.45. Found: C, 66.47; H, 4.98; N, 6.61.

Procedure for the synthesis of compound 9

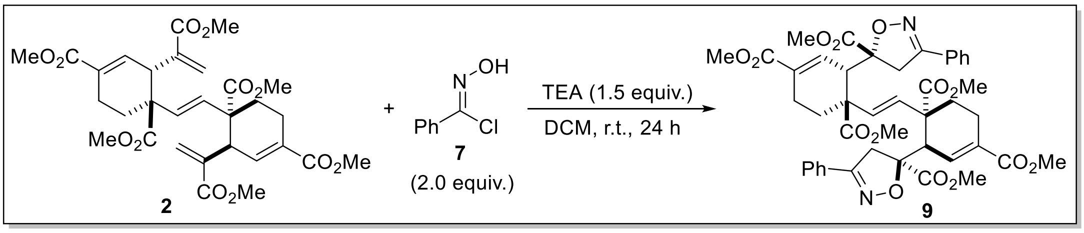

In a round bottom flask, triethylamine (1.5 equiv., 1.5 mmol, 151.8 mg) and the chloro-oxime **7** (2.0 equiv., 2.0 mmol, 310.0 mg) were added to the solution of the compound **2** (1.0 equiv., 1.0 mmol, 588.2 mg) in DCM dry (0.1 M). The resulted solution was magnetically stirred at room temperature for 24 hours. The reaction mixture was washed with brine (3 x 5 mL) and the organic layer was extracted with DCM (2 x 5 mL), dried over MgSO4 and filtered. The solvent was evaporated under reduced pressure. Compound **9** was obtained (462.7 mg, 56%) as white solid after FCC – AcOEt/hexane (3:7); m.p.: 215-216 °C. ^{1}H NMR ($CDCl_3$, 400 MHz) δ 7.66 (d, 2H, *J* = 8.1 Hz), 7.43-7.36 (m, 3H), 6.96 (d, 1H, *J* = 1.8), 5.93 (s, 1H), 4.09 (d, 1H, *J* = 3.4 Hz), 3.80 (d, 1H, *J* = 17.0 Hz), 3.77 (s, 3H), 3.70 (s, 3H), 3.66 (s, 3H), 3.37 (d, 1H, *J* = 17.1 Hz), 2.58-2.51 (m, 1H), 2.44-2.38 (m, 1H), 2.23-2.16 (m, 1H), 2.04-1.98 (m, 1H); ^{13}C NMR ($CDCl_3$, 101 MHz) δ 174.4, 171.5, 166.7, 157.2, 134.6, 131.9, 130.5, 129.5, 128.7, 128.6, 126.9, 90.0, 53.1, 52.6, 51.8, 49.4, 46.4, 41.8, 28.3, 21.7; IR ν_{max} 1713, 1650 cm^{-1}. Anal. Calcd. For $C_{44}H_{46}N_2O_{14}$: C, 63.92; H, 5.61; N, 3.39. Found: C, 64.12; H, 5.44; N, 3.22.

Computational methods

The structures of **VI**, **VII**-*RR*/*RS*, and **2**-*RRSS*/*RRRR*, were constructed with the MOE software.[5] Geometries were minimized with the MMFF94x force field,[6] and the Born solvation model. The dielectric constant was set to 8.9 to simulate dichloromethane (DCM). All geometries within the 1.5 kcal/mol interval were reoptimized by density functional theory (DFT) using the range-separated ωB97X-D functional, that includes empirical atom–atom dispersion corrections,[7] and the 6-31+G(d,p) basis set. Single point (SP) energy calculations were then done with the same functional, coupled to the 6-311++g(3df,3pd) basis set, and the CPCM solvation model for DCM.[8]

Concerning **2**-*RRSS*, it should be noted that the X-ray crystal structure of the pentaene **2** does not correspond to the global minimum. Indeed, the lowest energy geometry differs in the orientation of the two methoxycarbonyl moieties at position 4 of one cyclohexene ring and at position 1 of the other ring (Figure S3). Several trials were carried out using different DFT methods, but no one found the geometry of the X-ray crystal structure of **2** as the global minimum, probably due to supramolecular packing in the crystal. However, the global minimum differs from the X-ray geometry by 0.2 kcal/mol only (Table S1), well below the accuracy limit of the method,[9] and thus it was used as the reference structure for subsequent calculations.

The lowest energy structures of **VII**-*RR*, **VII**-*RS*, **2**-*RRSS*, and **2**-*RRRR* were used as starting point to locate the corresponding transition states (TS). Since a thorough conformational search is not possible for TSs, several conformations were evaluated by arbitrarily, but wisely, variating the dihedral of rotatable bonds, and the lowest energy structure was further considered for each TS. Intrinsic reaction coordinate (IRC) analyses were performed for each TS by tracing the reaction path in both the “forward” and “reverse” directions. Fifty points along the reaction path were computed for each IRC calculation, which was conducted using the same level of theory applied for geometry optimization. IRC paths confirmed that each TS connected the expected activated complexes (AC) and products. The ACs resulting from the IRC analyses were thoroughly optimized, and SP energies calculated as described above. Optimized geometries of TS-**VII** (*RR* and *RS*), and TS-**2** (*RRSS* and *RRRR*) are depicted in Figures S4 and S5, respectively. Results from IRC analyses are reported in Figures S6 and S7. The Gaussian16 software was used for geometry optimizations, SP energy calculations and IRC analyses.[10]

QTAIM calculations were performed using the Multiwfn software.[11] A wavefunction file was generated for TS-**VII**-*RR*/*RS* and TS-**2**-*RRSS*/*RRRR* by setting “output=wfn” in the Gaussian root section. The wavefunction was loaded into Multiwfn, and BCPs and BPs were computed by topology analysis. BCPs were searched by starting from nuclear positions, from midpoint of atomic pairs, from the triangle center of three atoms, and from the pyramid center of four atoms. BPs connecting (3,−3) and (3,−1) BCPs were then generated. Molecular graphs were subsequently generated, and BCPs connecting non-covalently bound oxygens and hydrogens (HB BCP) analyzed.

Table S1. Absolute energies, thermochemical corrections, and number of imaginary frequencies (#IF) for all the stationary points discussed here.

cmpd	E(SCF)	ZPEc	Hc	Gc	SP_Esol	Esol+ZPE	Esol+Hc	Esol+Gc	# IF
**VI**	-688.956399	0.207361	0.223501	0.162786	-689.178896	-688.971535	-688.955395	-689.016110	0
**AC-VII-*RR***	-1377.936023	0.416657	0.449480	0.350065	-1378.374577	-1377.957920	-1377.925097	-1378.024512	0
**AC-VII-*RS***	-1377.938228	0.417044	0.449572	0.352342	-1378.378298	-1377.961254	-1377.928726	-1378.025956	0
**TS-VII-*RR***	-1377.920813	0.418182	0.449115	0.355373	-1378.359614	-1377.941432	-1377.910499	-1378.004241	1
**TS-VII-*RS***	-1377.912104	0.417891	0.449178	0.353349	-1378.353037	-1377.935146	-1377.903859	-1377.999688	1

---

[5] Molecular Operating Environment (MOE), 2022.02 Chemical Computing Group ULC, 910-1010 Sherbrooke St. W., Montreal, QC H3A 2R7, Canada, 2023.
[6] Halgren, T. A. Merck molecular force field. I. Basis, form, scope, parameterization, and performance of MMFF94. *J. Comput. Chem.* **1996**, *17*, 490–519.
[7] Chai, J.-D.; Head-Gordon, M. Long-range corrected hybrid density functionals with damped atom–atom dispersion corrections. *Phys. Chem. Chem. Phys.* **2008**, *10*, 6615–6620.
[8] Cossi, M.; Rega, N.; Scalmani, G.; and Barone, V. Energies, structures, and electronic properties of molecules in solution with the C-PCM solvation model. *J. Comput. Chem.* **2003**, *24*, 669–681.
[9] Mardirossian, N.; Head-Gordon, M. Thirty years of density functional theory in computational chemistry: an overview and extensive assessment of 200 density functionals. *Mol. Phys.* **2017**, *115*, 2315–2372.
[10] Frisch, M. J. et al. (2016). Gaussian 16, Revision A. 03, Gaussian. Inc., Wallingford CT 3.
[11] Lu, T.; Chen, F. Multiwfn: A multifunctional wavefunction an-alyzer. *J. Comput. Chem.* **2012**, *33*, 580–592.

**VII-RR**	-1377.988432	0.422707	0.453269	0.359180	-1378.426591	-1378.003884	-1377.973322	-1378.067411	0
**VII-*RS***	-1377.988237	0.422681	0.453299	0.358907	-1378.428381	-1378.005700	-1377.975082	-1378.069474	0
**AC-2-*RRSS***	-2066.973559	0.632607	0.679734	0.547856	-2067.629730	-2066.997123	-2066.949996	-2067.081874	0
**AC-2-*RRRR***	-2066.975359	0.631912	0.679163	0.547977	-2067.631650	-2066.999738	-2066.952487	-2067.083673	0
**TS-2-*RRSS***	-2066.959137	0.633394	0.678897	0.552706	-2067.614151	-2066.980757	-2066.935254	-2067.061445	1
**TS-2-*RRRR***	-2066.949678	0.633814	0.679237	0.553274	-2067.604788	-2066.970974	-2066.925551	-2067.051514	1
**2-*RRSS***	-2067.033177	0.638232	0.683051	0.557642	-2067.688648	-2067.050416	-2067.005597	-2067.131006	0
**2-*RRSS* Xray**	-2067.032471	0.638355	0.682938	0.559058	-2067.688190	-2067.049835	-2067.005252	-2067.129132	0
**2-*RRRR***	-2067.031124	0.638294	0.683243	0.557126	-2067.687988	-2067.049694	-2067.004745	-2067.130862	0

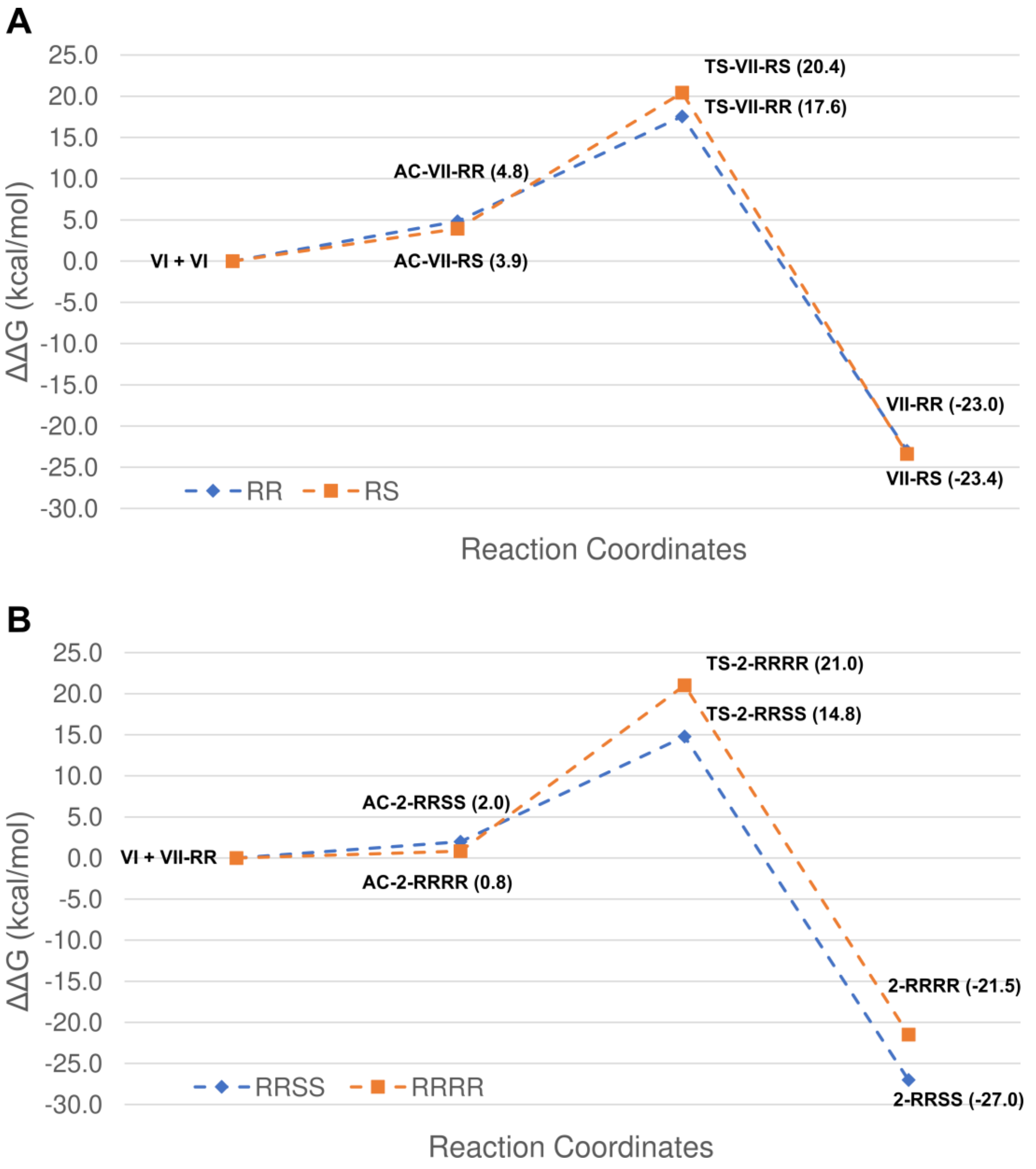

Figure S1. A. Gibbs free energy path for the dimerization of **VI**, leading to **VII**-*RS* and **VII**-*RR*. B. Gibbs free energy path for the addition of **VII**-*RR* to **VI**, leading to **2**-*RRRR* (not isolated) and **2**-*RRSS* (isolated) stereoisomers. *ΔG* values relative to the isolated reactants are reported in parentheses in kcal/mol.

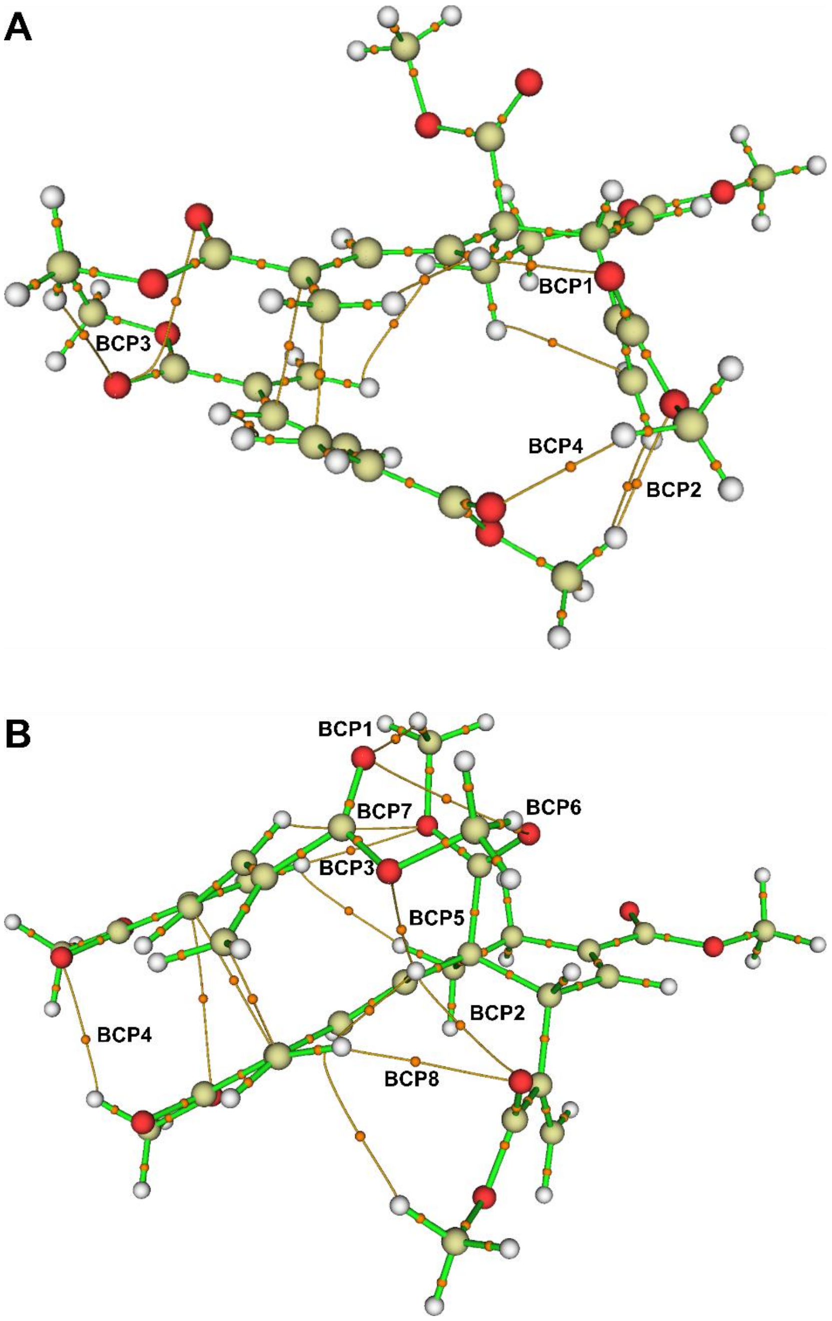

Figure S2. A and B. Bond paths and bond critical points found by QTAIM analysis for **2**-*RRSS* and **2**-*RRRR*, respectively.

Table S2. Hydrogen bonds (HB) BCPs and corresponding density ρ(r) (in a.u.) found for TS-**2**-*RRSS* and TS-**2**-*RRRR*. The sum of ρ(r) for all (3,-1) HB BCPs is also reported for each TS.

	TS-**2**-*RRSS*		TS-**2**-*RRRR*	
BCP	ρ(r)	Inter.[a]	ρ(r)	Inter.[a]
1	0.012831	Y	0.015344	N
2	0.010315	N	0.011318	Y
3	0.009550	Y	0.010954	Y
4	0.008226	Y	0.009994	Y
5	0.007720	Y		
6	0.007515	Y		
7	0.005814	Y		
8	0.004689	N		
$\sum\rho(r)_{HB}$	0.066659		0.047609	

[a] The BCP is considered “intermolecular” (Inter. = Y) if the corresponding BP connects the two reactants in the TSs.

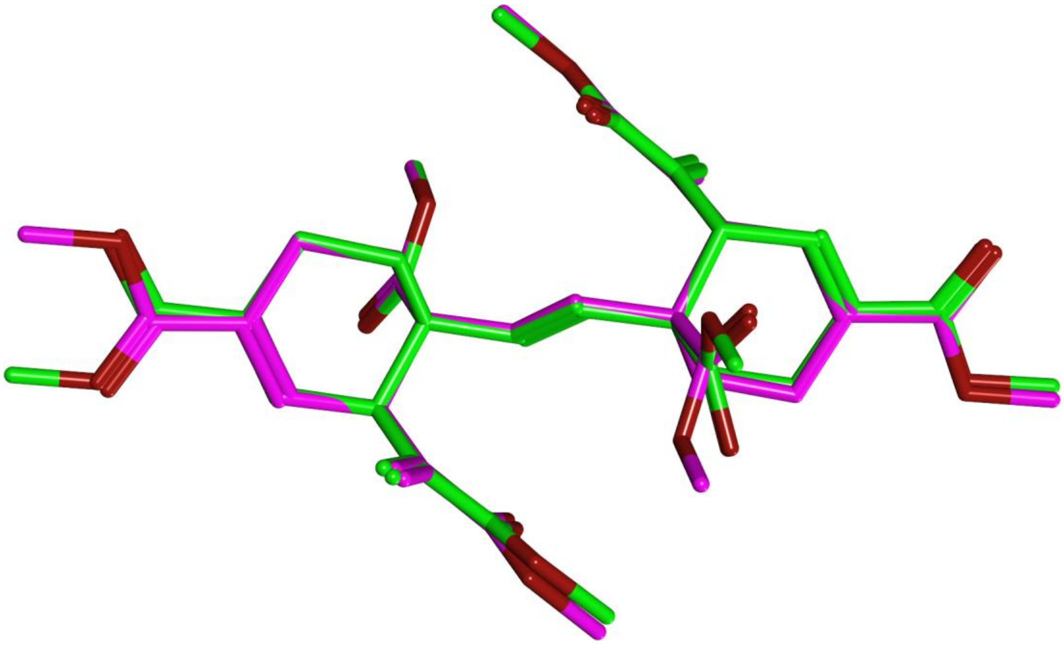

Figure S3. DFT optimized geometries of **2**-*RRSS* as found in the global minimum (green-colored carbons) and X-ray (magenta-colored carbons) structure.

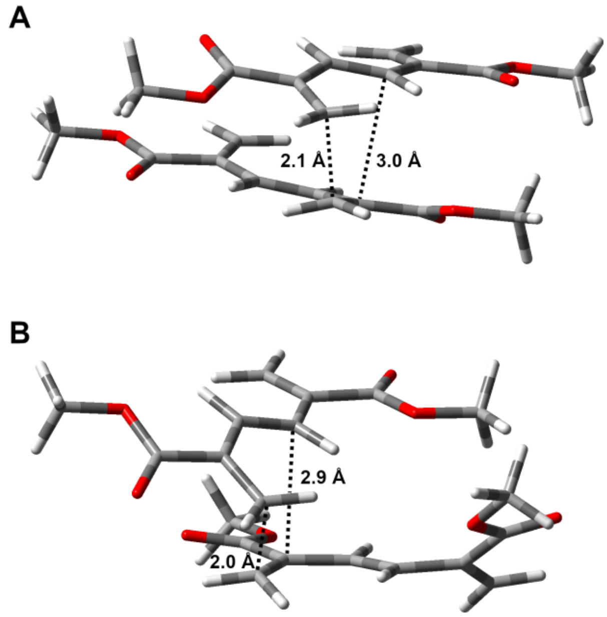

Figure S4. Optimized geometries and distances (in Angstrom) between reacting atoms of TS-**VII**-*RR* (A) and TS-**VII**-*RS* (B)

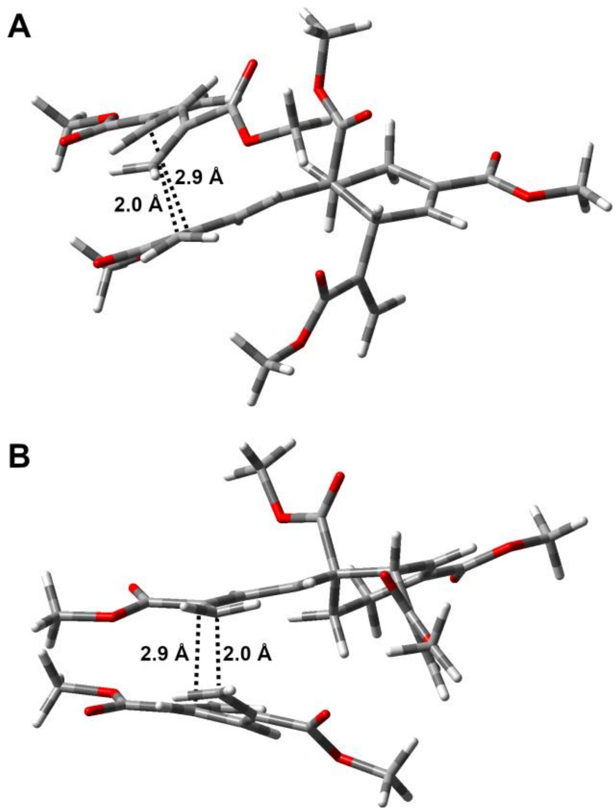

Figure S5. Optimized geometries and distances (in Angstrom) between reacting atoms of TS-**2**-*RRSS* (A) and TS-**2**-*RRRR* (B)

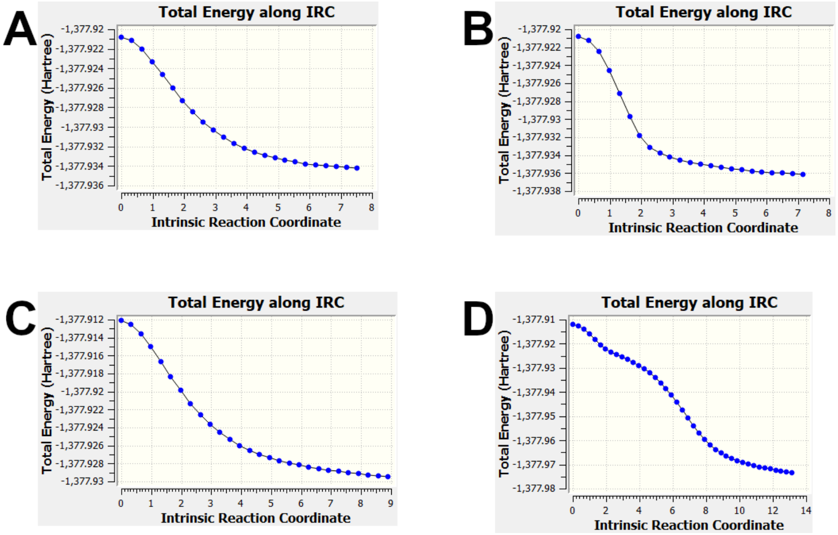

Figure S6. Results from IRC analyses starting from TS-**VII**-*RR* (A: from TS to activated complex; B from TS to product) and from TS-**VII**-*RS* (C: from TS to activated complex; D from TS to product).

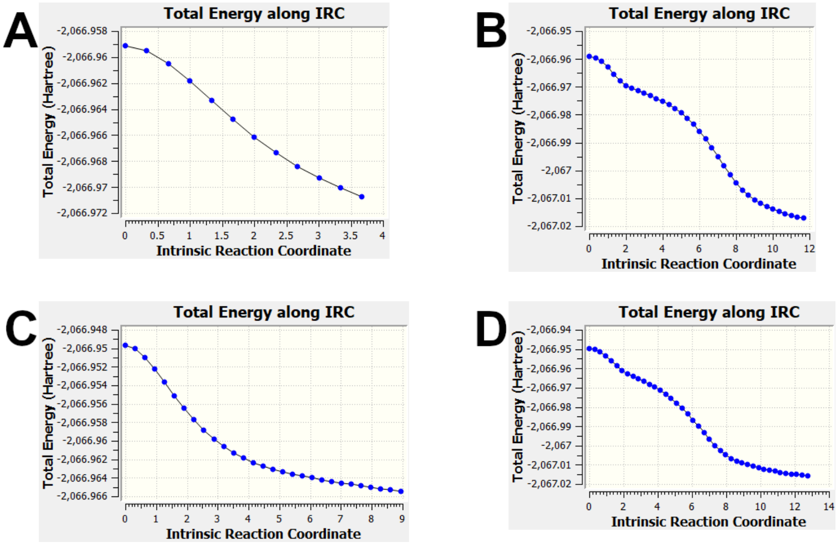

Figure S7. Results from IRC analyses starting from TS-**2**-*RRSS* (A: from TS to activated complex; B from TS to product) and from TS-**2**-*RRRR* (C: from TS to activated complex; D from TS to product).

Gaussian input and Cartesian coordinates of optimized geometries

Gaussian root for geometry optimization (ground states)
opt freq wb97xd/6-31+g(d,p) 10f 6d gfinput gfprint
Gaussian root for geometry optimization (transition states)
opt=(calcfc,ts,noeigentest) freq wb97xd/6-31+g(d,p) 10f 6d gfinput gfprint
Gaussian root for single point calculations
wb97xd/6-311++g(3df,3pd) scrf=(cpcm,solvent=ch2cl2) 10f 6d gfinput gfprint geom=checkpoint guess=read

VI
0 1

O	4.03760000	0.99465400	-0.15463200
O	-4.03761300	-0.99463200	-0.15462300
O	-2.93253600	0.89630900	-0.65063800
O	2.93254800	-0.89630200	-0.65063900
C	3.04194100	0.30960800	-0.07208700
C	1.79851700	0.68919500	0.67637700
C	1.82697800	1.84118600	1.35981600
C	-0.63547000	0.21339000	0.63574900
C	0.63546400	-0.21339800	0.63572200
C	-1.79852400	-0.68920700	0.67637400
C	-1.82698000	-1.84122800	1.35976200
C	-3.04195400	-0.30959000	-0.07206400
C	-4.07608800	1.33839000	-1.38539700
C	4.07610900	-1.33837500	-1.38538600
H	0.98338900	2.15409100	1.96593500
H	2.70680300	2.47467900	1.32698500
H	-0.84403500	1.27781800	0.56880300
H	0.84402100	-1.27782400	0.56870100
H	-0.98338800	-2.15415500	1.96586500
H	-2.70681000	-2.47471600	1.32691500
H	-4.94681600	1.40707700	-0.72968600
H	-4.29525100	0.64460300	-2.19979400
H	-3.80965700	2.31896100	-1.77660700
H	4.94684200	-1.40702900	-0.72967700
H	4.29525900	-0.64460300	-2.19979900
H	3.80970100	-2.31896100	-1.77657500

AC-VII-*RR*
0 1

O	-3.04777600	0.21490900	1.57897900
O	3.04773700	0.21248400	-1.57900300
O	3.83516800	-0.18319300	1.43138000
O	3.99901300	2.00789600	0.96580400
O	3.34067000	-2.00934200	-1.44656300
O	-3.34072400	-2.00708300	1.44932300
C	-2.58610600	-0.90814800	1.52882300
C	-1.11572000	-1.19971400	1.54962700
C	-0.67338500	-2.46544600	1.51857000
C	0.67339400	-2.46784100	-1.51602100
C	1.11567700	-1.20212500	-1.54851200
C	1.05664700	-0.00025200	1.82583000
C	-0.25250000	-0.01058300	1.54309200
C	0.25246600	-0.01298500	-1.54371600
C	2.58605800	-0.91051200	-1.52762500
C	4.75184400	-1.79724400	-1.34669100
C	1.90019900	1.19841100	1.70028500
C	1.47193100	2.45538800	1.88041700
C	3.33171300	0.92061000	1.35730900
C	5.33362300	1.79006600	0.50588300
C	-4.75193400	-1.79515200	1.34965400
C	-1.05678100	-0.00309500	-1.82601400
C	-1.90027100	1.19578600	-1.70217400
C	-1.47204300	2.45246900	-1.88444600
C	-3.33166700	0.91853600	-1.35825000
O	-3.83512100	-0.18539900	-1.43034000
O	-3.99886100	2.00645800	-0.96835300
C	-5.33333300	1.78937100	-0.50766700
H	0.38817400	-2.68835100	1.49302100
H	-1.36488900	-3.29905200	1.51049500
H	-0.38815700	-2.69079600	-1.49057000
H	1.36494500	-3.30139400	-1.50665800
H	1.57794500	-0.91119400	2.10733300
H	-0.74653900	0.90778100	1.23660500
H	0.74662700	0.90587300	-1.23891300
H	5.19442400	-2.79229600	-1.35672100
H	4.97851000	-1.28272900	-0.41050000
H	5.10967200	-1.21024900	-2.19589900
H	0.45110400	2.65748300	2.18920000
H	2.13610700	3.29878000	1.73533700
H	5.30892100	1.18677300	-0.40445800
H	5.92907700	1.28860100	1.27198400
H	5.73392800	2.78065600	0.29526800
H	-4.97897600	-1.28200600	0.41279600
H	-5.10946300	-1.20693600	2.19814100
H	-5.19448900	-2.79019800	1.36129200
H	-1.57824000	-0.91449500	-2.10572400
H	-2.13617000	3.29609900	-1.74053600
H	-0.45130500	2.65405700	-2.19385400
H	-5.92903500	1.28673900	-1.27281100
H	-5.73354400	2.78029800	-0.29846500
H	-5.30835300	1.18748700	0.40359500

AC-VII-*RS*
0 1

O	3.46854100	-2.78717200	-0.24980000
O	3.02116100	1.59107000	1.43479100
O	-1.73639300	3.39694200	-0.86215400
O	-2.45237500	1.28543000	-0.59730500
O	0.90373400	2.24178900	1.04934300
O	3.74670300	-0.60520700	-0.70741100
C	3.04167300	-1.74076600	-0.68972100
C	1.67009400	-1.55237400	-1.27194600
C	0.93895300	-2.65547400	-1.47728800
C	2.02492800	-0.91070800	2.29476500
C	1.20854200	0.07884300	1.91385400
C	-0.06024500	0.21644900	-1.54548200
C	1.22007900	-0.18237800	-1.56295500
C	-0.25960600	-0.02708500	1.92747700
C	1.83078300	1.36295000	1.45287300
C	1.39015300	3.48795000	0.54107100
C	-0.45468600	1.60673100	-1.81984500
C	0.14725500	2.39396600	-2.72136900
C	-1.59026900	2.20687800	-1.04636200
C	-3.57607100	1.79424600	0.13325100
C	5.04980400	-0.65065100	-0.12565100
C	-0.93629200	-1.10756100	1.51897200
C	-2.40498200	-1.18646100	1.59990500
C	-3.10389900	-0.81576600	2.68039600
C	-3.18203100	-1.66705400	0.41413600
O	-4.38584700	-1.57997400	0.29644300
O	-2.39556700	-2.20343600	-0.53135800
C	-3.06849500	-2.63149800	-1.71870100
H	-0.04713700	-2.59685200	-1.92308900
H	1.32787900	-3.62922100	-1.20008100
H	3.10040600	-0.77871600	2.25323400
H	1.63447500	-1.85527500	2.65729700
H	-0.84572500	-0.47207600	-1.24760200
H	2.00233600	0.55167100	-1.73325100

H	-0.81133800	0.85201500	2.25246300
H	1.90549200	4.04119900	1.32941200
H	0.50603700	4.02121200	0.19481000
H	2.08084600	3.30938200	-0.28594300
H	0.93945200	2.01229000	-3.35703500
H	-0.15895500	3.42759800	-2.84140600
H	-3.23678500	2.24480900	1.06876200
H	-4.21388700	0.93376200	0.32707600
H	-4.10231900	2.54545200	-0.45856800
H	5.00262800	-1.06481400	0.88319500
H	5.38873900	0.38269300	-0.09460300
H	5.71372300	-1.26735600	-0.73613200
H	-0.40587000	-1.93730200	1.05641500
H	-4.18825500	-0.85164200	2.67402300
H	-2.59927500	-0.48770300	3.58290800
H	-3.83309800	-3.37282600	-1.47921600
H	-2.29876500	-3.06854500	-2.35349700
H	-3.53439400	-1.77671500	-2.21383800

TS-VII-*RR*

0 1

O	2.65637200	-0.06221600	-2.07081500
O	-2.65643600	-0.06213200	2.07082200
O	-4.24227800	-0.01442300	-1.07329800
O	-4.37624900	2.20420700	-0.78275100
O	-2.81420600	-2.17485300	1.31230700
O	2.81425400	-2.17491500	-1.31226500
C	2.12807900	-1.06641700	-1.63482100
C	0.66501800	-1.21813100	-1.41092600
C	0.12515300	-2.43751700	-1.02822000
C	-0.12508200	-2.43740700	1.02850900
C	-0.66500500	-1.21800800	1.41109100
C	-1.44778000	0.03022200	-1.23340000
C	-0.12418000	-0.02694500	-1.51817700
C	0.12414800	-0.02678100	1.51830400
C	-2.12808200	-1.06632900	1.63489100
C	-4.23329500	-2.10641800	1.48597700
C	-2.21808300	1.26659400	-1.09122400
C	-1.69430500	2.49918600	-0.99454100
C	-3.70014400	1.06904800	-0.98576600
C	-5.78930800	2.06620200	-0.62572600
C	4.23332700	-2.10645000	-1.48605000
C	1.44776200	0.03041200	1.23360900
C	2.21809100	1.26677300	1.09145500
C	1.69440900	2.49943900	0.99518800
C	3.70011600	1.06912900	0.98561500
O	4.24220200	-0.01437500	1.07305100
O	4.37624200	2.20423500	0.78238300
C	5.78925100	2.06612900	0.62499100
H	-0.93264800	-2.61224000	-1.19232800
H	0.75638600	-3.31754400	-1.07273200
H	0.93272100	-2.61207700	1.19266300
H	-0.75628400	-3.31745200	1.07308700
H	-2.02655400	-0.87809000	-1.10320500
H	0.43316500	0.87924800	-1.74149400
H	-0.43325600	0.87940100	1.74151900
H	-4.60049700	-3.10396700	1.24823500
H	-4.65624200	-1.36954200	0.80080400
H	-4.47610800	-1.84283100	2.51769900
H	-0.62112600	2.65741200	-1.02976500
H	-2.32841700	3.36951600	-0.88197300
H	-6.01296300	1.45005000	0.24804600
H	-6.23079300	1.60921000	-1.51397200
H	-6.16635000	3.07806000	-0.48574300
H	4.65630700	-1.36953900	-0.80093600
H	4.47605100	-1.84289200	-2.51780000
H	4.60057300	-3.10398200	-1.24830400
H	2.02655800	-0.87789100	1.10345100
H	2.32858300	3.36972300	0.88261500
H	0.62126200	2.65779100	1.03078800
H	6.23093800	1.60913500	1.51313500
H	6.16632400	3.07795600	0.48487600
H	6.01263000	1.44993500	-0.24882100

TS-VII-*RS*

0 1

O	-4.01744200	-2.77240500	-0.17525700
O	-2.58077900	1.08052800	-1.80367100
O	1.36116900	3.44617200	1.81082500
O	1.87773300	1.27909300	1.54130200
O	-0.69265500	2.30795700	-1.84438400
O	-4.47854100	-1.08446000	1.23387900
C	-3.64577900	-1.82734400	0.49094600
C	-2.22965300	-1.37027800	0.57319500
C	-1.29786500	-2.14115400	-0.10585500
C	-1.08569800	-1.27406900	-1.91946100
C	-0.47173800	-0.03640800	-1.77217900
C	-0.69023300	0.47464900	1.06764600
C	-1.92736900	-0.09406900	1.12405500
C	0.96256000	0.13350200	-1.60519100
C	-1.36418800	1.14242100	-1.79867200
C	-1.49280000	3.48735000	-1.91365300
C	-0.41179600	1.86853600	1.42464200
C	-1.34443200	2.82629000	1.54540200
C	1.01521700	2.30135600	1.61427500
C	3.26012900	1.62080400	1.69433100
C	-5.85832900	-1.45023500	1.17113200
C	1.85037800	-0.86347100	-1.42733300
C	3.27416100	-0.62360800	-1.15631600
C	4.00031900	0.35731300	-1.71037400
C	3.96858400	-1.51256500	-0.16844800
O	5.06697500	-1.30285100	0.30093200
O	3.22727800	-2.58144400	0.16130900
C	3.81375900	-3.47737200	1.10902100
H	-0.24375500	-2.04579500	0.14258000
H	-1.62193000	-3.12121400	-0.44156400
H	-2.12775500	-1.28760200	-2.21861000
H	-0.48578300	-2.10611700	-2.27547900
H	0.17367500	-0.12112200	0.80457100
H	-2.76520500	0.47670900	1.50986000
H	1.30993400	1.15964700	-1.52878800
H	-2.05401000	3.51093700	-2.85090000
H	-0.79114100	4.31877700	-1.86459600
H	-2.19229700	3.52817000	-1.07659700
H	-2.39665500	2.62220400	1.37807300
H	-1.05093400	3.83794500	1.80226000
H	3.56833900	2.30266900	0.89900600
H	3.80956100	0.68423100	1.62184000
H	3.42581000	2.09717600	2.66272800
H	-6.22805400	-1.35867700	0.14762900
H	-6.37431400	-0.75350500	1.82991800
H	-5.99648200	-2.47838200	1.51277200
H	1.52936500	-1.90085200	-1.41679800
H	5.03545600	0.50346400	-1.42122800
H	3.57370100	1.01254400	-2.46248800
H	4.75286100	-3.87914700	0.72291000
H	3.08317500	-4.27246700	1.24884100
H	4.00517500	-2.96019200	2.05160400

VII-*RR*

0 1

O	-1.93668600	3.81749700	-1.21404800
O	2.14453600	-0.57827000	2.81814600
O	4.07858600	1.13265900	0.34383000
O	4.30824300	0.19523300	-1.68162000
O	2.57359700	-1.46314500	0.80044900
O	-3.29923200	2.98360300	0.36503300
C	-2.12210900	3.06568000	-0.27982400
C	-1.11454700	2.12676900	0.29085000
C	-1.48310500	1.24714200	1.45340800
C	-0.25885200	0.53894600	2.03406800
C	0.68418600	-0.01779100	0.95112700
C	1.23233400	1.22040500	0.14478300
C	0.09522500	2.11234900	-0.27837700
C	0.01374500	-1.02301800	0.03564300
C	1.86544400	-0.70193000	1.64965900

C	3.76104400	-2.04955800	1.33957300
C	2.13300000	0.87034100	-1.02367200
C	1.70678200	0.72413300	-2.28118400
C	3.58765700	0.75130800	-0.69897600
C	5.71364300	0.10197700	-1.44145600
C	-4.33169700	3.84305700	-0.11973000
C	-1.04175300	-1.77527200	0.35379400
C	-1.61918200	-2.77894300	-0.56023500
C	-0.88930700	-3.60719500	-1.31710900
C	-3.11097700	-2.88736800	-0.65363200
O	-3.71171000	-3.65935700	-1.36800500
O	-3.72506800	-2.00879400	0.15653500
C	-5.15357800	-2.03146200	0.12945000
H	-1.95313600	1.85278300	2.23456600
H	-2.24582800	0.52961900	1.13059500
H	-0.56793200	-0.26207500	2.71127100
H	0.31896600	1.23643300	2.64736100
H	1.85605000	1.78835300	0.84860400
H	0.28558900	2.80534900	-1.09410300
H	0.46122000	-1.14722400	-0.94691300
H	4.19759500	-2.62295600	0.52250000
H	3.51727000	-2.70230500	2.18014100
H	4.44326000	-1.26366300	1.67291200
H	0.65792500	0.84790400	-2.53527100
H	2.39501100	0.48350900	-3.08286000
H	5.90999500	-0.51484000	-0.56147800
H	6.14235300	1.09404400	-1.28446400
H	6.13145900	-0.36067200	-2.33421500
H	-4.55672100	3.61766300	-1.16462100
H	-4.02735900	4.88893500	-0.03879600
H	-5.19705300	3.64520700	0.51130500
H	-1.54055200	-1.65821700	1.31142600
H	-1.37497100	-4.30627600	-1.98956400
H	0.19438400	-3.60127000	-1.26229200
H	-5.51776700	-1.81253800	-0.87660100
H	-5.47041100	-1.25996500	0.82960100
H	-5.52521100	-3.01063800	0.43937400

VII-*RS*

0 1

O	2.51319000	3.53695500	0.72224000
O	-1.75551300	-1.88072100	2.27491400
O	-4.89918200	0.13458800	0.24732100
O	-3.68438600	0.07403300	-1.64086500
O	-2.31439500	-2.03553400	0.10523000
O	1.09842800	4.01027500	-0.95681800
C	1.52408500	3.26167700	0.07441800
C	0.67484600	2.06827500	0.34276400
C	1.11167800	1.20629200	1.49432600
C	0.05630400	0.16985500	1.87416100
C	-0.57995600	-0.49999300	0.64752100
C	-1.27297500	0.58462000	-0.24436900
C	-0.38640200	1.78692600	-0.42225700
C	0.42445900	-1.25735000	-0.20964600
C	-1.60471300	-1.53222300	1.12891300
C	-3.32378700	-2.98916000	0.45475700
C	-2.64984400	0.99062800	0.26721200
C	-2.85994700	1.89246500	1.22869100
C	-3.86099800	0.34999000	-0.34004000
C	-4.78845200	-0.54736400	-2.29870700
C	1.88886600	5.16208400	-1.25291100
C	1.58226700	-1.76486200	0.21695100
C	2.50815900	-2.51325600	-0.65417200
C	2.12358700	-3.46010200	-1.51758100
C	3.97095300	-2.19363000	-0.57327400
O	4.83911500	-2.73031800	-1.22530100
O	4.21316200	-1.21693300	0.31473300
C	5.57989900	-0.82067400	0.45312200
H	2.06048300	0.72665300	1.22774300
H	1.33640800	1.84202600	2.35634900
H	-0.74586800	0.64106800	2.45036500
H	0.48050400	-0.58652300	2.53825700
H	-1.42860900	0.14626800	-1.23383300
H	-0.65867100	2.46176900	-1.22834000
H	0.14487800	-1.39252800	-1.25355200
H	-4.11625300	-2.49866900	1.02480300
H	-2.89398500	-3.80174900	1.04324000
H	-3.70995100	-3.36316100	-0.49263100
H	-2.03785500	2.44041500	1.67790200
H	-3.86625200	2.10480000	1.57370000
H	-5.03103800	-1.49733000	-1.81671200
H	-4.46413700	-0.70983000	-3.32535500
H	-5.66661200	0.10134800	-2.26961200
H	1.39940900	5.64149000	-2.09948000
H	2.90846500	4.86953600	-1.51379800
H	1.91985700	5.83649000	-0.39418600
H	1.90849800	-1.60948500	1.24110600
H	2.85433800	-3.95582700	-2.14780200
H	1.08246300	-3.75627500	-1.59264500
H	5.57623000	-0.01507100	1.18544900
H	5.97082400	-0.46932000	-0.50398400
H	6.18534000	-1.66005400	0.80209900

AC-2-*RRSS*

0 1

O	0.64816800	-0.65591400	-3.82725200
O	-5.18610300	3.62135900	0.72379100
O	3.96623600	-1.95340600	1.97010200
O	-2.21060100	0.26978700	-2.36229500
O	5.77013300	1.51085800	0.29480200
O	-1.86334100	-3.32994500	0.11031600
O	4.82806000	3.52812100	0.57598000
O	-1.76658200	-3.48521500	2.34641900
O	2.88998800	-0.04696000	2.46662100
O	-0.64672600	1.72285200	-1.67486800
O	0.50988200	-2.66655300	-2.83502500
O	-6.31931800	1.71504400	0.36612700
C	1.10768600	-1.49743300	-3.08325400
C	-5.19078000	2.41949600	0.55020900
C	2.37858900	-1.31602100	-2.31282000
C	-3.95433800	1.59223700	0.52175900
C	3.18635300	-2.36211900	-2.09365000
C	-2.66588500	2.34342500	0.73689100
C	1.68872000	-2.86156300	0.62664600
C	-1.47951200	1.40565400	0.95612100
C	1.72670400	-1.63224900	1.15985600
C	-1.49230700	0.22617600	-0.02662700
C	3.44846200	0.43566100	-0.88755300
C	-2.79137300	-0.61311300	0.20737500
C	2.59472400	0.05910800	-1.84920700
C	-4.00215600	0.27568800	0.28742600
C	0.63168800	-0.65207800	1.05252800
C	-0.27909500	-0.67165600	0.07612200
C	2.97546000	-1.25738500	1.89561000
C	-1.51954400	0.72742100	-1.48138800
C	4.06555800	0.40136100	3.14741800
C	-0.56335800	2.23795800	-3.01284000
C	3.48513300	1.80315900	-0.34793000
C	-2.68981500	-1.50712800	1.43171300
C	2.40611000	2.59563800	-0.26176000
C	-3.14142200	-1.17140300	2.64343400
C	4.80732200	2.24308300	0.19302100
C	-2.06720100	-2.85043900	1.20805700
C	6.06650200	3.99259500	1.11613700
C	-1.19592000	-4.78818000	2.20836400
C	-0.76341300	-2.86379800	-3.46910200
C	-7.52964700	2.47274600	0.37070500
H	2.91847400	-3.35456300	-2.43781600
H	-2.77791700	3.01855400	1.59061300
H	4.13528800	-2.24735500	-1.57943600
H	-2.48530800	2.99629200	-0.12615200
H	2.54360900	-3.52114100	0.73071300
H	-0.54306000	1.96149500	0.86835900
H	0.81942700	-3.21971400	0.08382400
H	-1.51794300	0.99605500	1.97174800
H	4.15050700	-0.26946200	-0.44910400

H	-2.91395800	-1.26821600	-0.66044700
H	1.93871500	0.79995300	-2.29854700
H	-4.96472600	-0.20700100	0.14865400
H	0.59666900	0.11022400	1.82357400
H	-0.21455300	-1.43289900	-0.69918200
H	4.27758700	-0.24341100	4.00336600
H	0.19227900	3.02187400	-2.96984500
H	3.83838600	1.41443900	3.47772200
H	-0.26401800	1.44377400	-3.69811800
H	4.92146300	0.39688600	2.46904700
H	-1.52818000	2.65188000	-3.31354300
H	1.42963300	2.24080300	-0.57970200
H	-3.63224600	-0.21760500	2.80999400
H	2.48165400	3.59847300	0.14197200
H	-3.03794100	-1.84427400	3.48637200
H	5.90382600	5.04223600	1.35606700
H	-0.22688400	-4.72590400	1.70883500
H	6.86800600	3.88477800	0.38226000
H	-1.07398100	-5.16033000	3.22427300
H	6.32744000	3.42857500	2.01472100
H	-1.85975200	-5.43862700	1.63499400
H	-1.14495400	-3.79665700	-3.05897600
H	-7.65936500	2.98227600	1.32823700
H	-1.43251500	-2.03411200	-3.23005100
H	-8.32702300	1.74852000	0.21027500
H	-0.63790600	-2.93253800	-4.55185400
H	-7.51855200	3.21545200	-0.43017600

AC-2-*RRRR*
0 1

O	-5.59340300	-1.13186600	-2.36312900
O	-3.47196100	-1.33902500	2.78484900
O	-0.89490600	2.11438600	2.82526100
O	0.17473100	3.20937900	1.18813400
O	-2.49146900	-3.02304900	1.67602900
O	-6.05567900	0.77594000	-1.27128100
C	-5.28466200	-0.28944000	-1.54480300
C	-4.02373400	-0.33775300	-0.75605000
C	-3.12635200	-1.51470800	-1.03796900
C	-1.75009500	-1.36491700	-0.39177500
C	-1.84015600	-0.82208800	1.04051000
C	-2.49881900	0.59600300	1.01039100
C	-3.73759400	0.59799200	0.15702700
C	-0.50351500	-0.74359300	1.74797700
C	-2.71358500	-1.72696900	1.92592800
C	-3.20423400	-3.94818500	2.50073000
C	-1.53363000	1.68195200	0.55807000
C	-1.39981800	2.07821600	-0.71108800
C	-0.73263400	2.33607900	1.64246200
C	0.94509300	3.88309600	2.19285600
C	-7.28277400	0.85452900	-1.99711100
C	0.68316200	-0.96627400	1.18206000
O	2.79375400	4.69636900	-0.39687000
O	2.90564600	-2.63402900	0.73886600
O	4.39231500	-2.29700000	-2.01246800
O	2.50494400	-3.47959100	-2.30019400
O	4.30700100	-1.13282500	1.65035600
O	1.46767800	3.59870300	-1.83706500
C	2.40150200	3.65219800	-0.87442600
C	2.88673100	2.29205200	-0.48700200
C	3.88347400	2.21332200	0.40335100
C	2.18120400	0.16091400	2.82211300
C	1.97600900	-0.73879100	1.85117700
C	2.92861800	0.07795000	-1.58829800
C	2.24193700	1.11432900	-1.09210300
C	3.08881800	-1.60411700	1.35499300
C	5.40839300	-1.93494900	1.20084400
C	2.31400000	-1.12570700	-2.16963400
C	1.09998700	-1.16712800	-2.73466100
C	3.18596900	-2.34460000	-2.14495200
C	3.27069000	-4.68061900	-2.18061200
C	0.92013500	4.85679000	-2.23745500
H	-3.61640400	-2.43014200	-0.68474300

H	-3.02457100	-1.64174500	-2.12024200
H	-1.14257500	-0.66284800	-0.97368800
H	-1.22806100	-2.32421600	-0.40225400
H	-2.79587300	0.83334500	2.03632700
H	-4.42323300	1.42686500	0.30453200
H	-0.55943500	-0.42857100	2.78833200
H	-2.89794000	-4.93624800	2.16114300
H	-4.28145000	-3.81466600	2.37899100
H	-2.94048500	-3.80259300	3.55046100
H	-2.00585500	1.63710300	-1.49673400
H	-0.68474500	2.84312000	-0.98942800
H	1.56562500	4.59577300	1.65334000
H	1.57148300	3.16442400	2.72574000
H	0.28395400	4.38535500	2.90142300
H	-7.90730800	-0.01680300	-1.78759600
H	-7.09041300	0.90545900	-3.07125800
H	-7.76821800	1.76571600	-1.65048800
H	0.74905100	-1.32687200	0.15992300
H	4.24389800	1.25111000	0.75366700
H	4.33263800	3.11835100	0.79936900
H	1.37171900	0.79124600	3.17898300
H	3.15743800	0.27785900	3.27835800
H	4.01529700	0.08345800	-1.55544500
H	1.15419000	1.11643300	-1.11669800
H	5.38085600	-2.91438300	1.68400900
H	6.30200900	-1.38853300	1.49907200
H	5.36640700	-2.05935500	0.11685300
H	0.50035500	-0.26733300	-2.83510900
H	0.69225600	-2.09298800	-3.12258700
H	2.56057800	-5.49305800	-2.32611900
H	3.71638600	-4.73240100	-1.18501700
H	4.05598900	-4.71309500	-2.93893600
H	0.20677300	4.62643400	-3.02765200
H	1.70702600	5.51585000	-2.60931500
H	0.41872000	5.33529500	-1.39241500

TS-2-*RRSS*
0 1

O	0.49555600	1.14258400	3.90012300
O	-5.26560400	-3.45518900	-0.46382000
O	4.21280400	1.48444000	-1.85490500
O	-2.34990600	0.31655900	2.30856300
O	5.47080600	-1.65984400	-0.08652000
O	-1.58691200	3.34637200	-0.76106100
O	4.42744800	-3.63606600	-0.28072300
O	-1.10947300	2.97195300	-2.92106100
O	3.04340600	-0.37681700	-2.32910300
O	-0.92967900	-1.39914500	2.04369700
O	0.37795400	2.99556100	2.62960000
O	-6.30723500	-1.46720200	-0.55899800
C	0.93236700	1.83377800	3.00167600
C	-5.21217400	-2.24261500	-0.50064100
C	2.11279700	1.47622500	2.17173800
C	-3.93498500	-1.47895200	-0.48620800
C	2.81427100	2.45438300	1.48340900
C	-2.68123000	-2.31348000	-0.43578400
C	2.06578600	2.52277400	-0.41045100
C	-1.41959400	-1.48394600	-0.67346200
C	2.01365800	1.24882100	-0.95760000
C	-1.45851800	-0.14965700	0.08276000
C	3.23859600	-0.43913100	1.10834000
C	-2.67125300	0.68998000	-0.43956700
C	2.34324200	0.08253200	1.98794200
C	-3.92334500	-0.14076500	-0.48941300
C	0.85913800	0.38602500	-0.82090900
C	-0.19721300	0.67428800	-0.04036300
C	3.19825600	0.82219000	-1.74078900
C	-1.65682700	-0.37018100	1.59295500
C	4.16124700	-0.83538300	-3.09367800
C	-1.02388400	-1.66906000	3.45193600
C	3.22113000	-1.84604200	0.70738700
C	-2.38146700	1.32897700	-1.78829800
C	2.14152500	-2.64140200	0.80262600

C	-2.74960300	0.80561500	-2.96090200
C	4.48603700	-2.34749800	0.08542700
C	-1.66195300	2.64203200	-1.74793600
C	5.61084100	-4.16154000	-0.88403500
C	-0.40560300	4.21518500	-2.95231300
C	-0.83660800	3.35297000	3.30385000
C	-7.55627000	-2.15925400	-0.56196900
H	2.62924800	3.49403800	1.73155300
H	-2.75098900	-3.11601200	-1.17637300
H	3.81866700	2.22797000	1.13935100
H	-2.62942400	-2.82004300	0.53570700
H	2.86065700	3.17247200	-0.76331700
H	-0.53842000	-2.06167800	-0.38590000
H	1.14047200	3.00855100	-0.11709000
H	-1.32101000	-1.25763900	-1.74114200
H	4.03991100	0.16177900	0.69095500
H	-2.83365600	1.50327000	0.27464800
H	1.65005800	-0.57894900	2.50000000
H	-4.86258000	0.40174600	-0.53778500
H	0.89224500	-0.54818200	-1.37085200
H	-0.23488600	1.61446500	0.50327000
H	4.34453400	-0.16661700	-3.93794000
H	-0.37695800	-2.52919300	3.62106900
H	3.88029700	-1.82723800	-3.44711900
H	-0.67918000	-0.80412000	4.01973000
H	5.05421300	-0.88288300	-2.46677600
H	-2.05615800	-1.90858100	3.71584300
H	1.19950400	-2.26461400	1.19078200
H	-3.30944000	-0.12333900	-3.00521000
H	2.18290200	-3.67547000	0.48228300
H	-2.50723200	1.29798600	-3.89525700
H	5.38848800	-5.20528100	-1.10064300
H	0.44397000	4.18467500	-2.26637300
H	6.45755600	-4.08168500	-0.19895500
H	-0.05952200	4.32724700	-3.97850500
H	5.84383000	-3.61958200	-1.80364500
H	-1.06680400	5.03861800	-2.67435700
H	-1.17208500	4.26914000	2.82124800
H	-7.62418100	-2.82377900	-1.42631600
H	-1.57602300	2.55827200	3.18320900
H	-8.31882500	-1.38343800	-0.61353100
H	-0.64375800	3.51969700	4.36584400
H	-7.66891800	-2.74847700	0.35088000

TS-2-*RRRR*

0 1

O	-4.99049400	-3.31371000	-1.69150500
O	-2.19481600	-1.63107400	2.86169800
O	-1.86826900	2.67072000	2.06718400
O	-2.50852300	3.89859300	0.30356000
O	-0.78959700	-2.81979300	1.57974700
O	-6.16958200	-1.81335500	-0.50666200
C	-5.02242800	-2.34093400	-0.96501600
C	-3.80339000	-1.62417800	-0.50255600
C	-2.49584300	-2.19705500	-0.98437000
C	-1.29793100	-1.30988100	-0.64298600
C	-1.41296300	-0.67084100	0.75225400
C	-2.69625400	0.20474800	0.81990900
C	-3.88694100	-0.56153100	0.30694000
C	-0.16835800	0.11494100	1.14054700
C	-1.54328000	-1.74630900	1.84920200
C	-0.72319500	-3.81181800	2.60697200
C	-2.60807300	1.53974700	0.08781500
C	-2.86061500	1.68374600	-1.21661000
C	-2.28014200	2.73778600	0.92685100
C	-2.16904600	5.08032800	1.04207000
C	-7.36431500	-2.47720000	-0.91908300
C	1.07683800	-0.38194600	1.05517300
O	0.68776200	4.45116300	-0.28713500
O	3.51670600	-1.75055300	1.44223400
O	5.33238400	-1.24498900	-1.27908300
O	4.18784500	-3.05587800	-1.94325200
O	4.61074500	0.17992200	1.82150700
O	0.28802600	3.28569500	-2.16590600
C	0.94003900	3.50719500	-1.01292500
C	2.02391300	2.52512800	-0.76608000
C	2.88706900	2.80239700	0.28485100
C	2.32134600	1.63338200	1.86689300
C	2.28377300	0.30181000	1.48111200
C	3.04080500	0.36097200	-1.29061600
C	2.08368200	1.30575100	-1.50063100
C	3.50274100	-0.54100600	1.57161200
C	5.83037700	-0.56398000	1.91694700
C	3.01632200	-0.99726200	-1.83016000
C	1.95468200	-1.58262900	-2.40733500
C	4.29588000	-1.75749000	-1.65047900
C	5.36348700	-3.83908200	-1.72858600
C	-0.66204700	4.28394900	-2.54291200
H	-2.36440300	-3.19249500	-0.54432100
H	-2.54963400	-2.36396900	-2.06501400
H	-1.20552300	-0.48875500	-1.36340700
H	-0.38119300	-1.89693600	-0.71060700
H	-2.86926400	0.42793800	1.87668200
H	-4.86375500	-0.19762300	0.60994200
H	-0.35155100	1.06904300	1.62453600
H	-0.06028500	-4.58474000	2.22174600
H	-1.71673600	-4.21760100	2.80934100
H	-0.31558100	-3.37857000	3.52281500
H	-3.13720000	0.83238900	-1.83016400
H	-2.81412300	2.65360700	-1.69494400
H	-2.47317200	5.91136700	0.40717400
H	-1.09280900	5.10999200	1.21629000
H	-2.71048600	5.09972200	1.98973100
H	-7.36277000	-3.51476100	-0.57751800
H	-7.45624700	-2.46029300	-2.00753200
H	-8.17985200	-1.92305200	-0.45655400
H	1.23680000	-1.38342700	0.66456700
H	3.87908400	2.36494900	0.29170300
H	2.79502100	3.77244100	0.76303100
H	1.38571500	2.15210000	2.04908600
H	3.16334300	1.96703900	2.46290600
H	3.94065300	0.60990900	-0.73985700
H	1.26404400	1.10637500	-2.18427800
H	5.75449400	-1.31390000	2.70753300
H	6.59735100	0.17066800	2.15891000
H	6.04638700	-1.04778900	0.96273700
H	1.01637800	-1.05204500	-2.52821700
H	2.00516700	-2.60248100	-2.76799600
H	5.09458400	-4.85290100	-2.02082700
H	5.64639300	-3.80577100	-0.67417400
H	6.18905600	-3.46722300	-2.33928300
H	-1.12115100	3.91660300	-3.46011000
H	-0.15443400	5.23405700	-2.72743300
H	-1.40858200	4.41977100	-1.75842400

2-*RRSS* (global minimum)

0 1

O	6.90907800	0.97450000	0.25577500
O	-6.70398100	0.92502700	1.04461500
O	2.56225500	-2.49749100	0.89167600
O	-2.74436000	0.98488000	-2.60007600
O	0.53189100	2.57951300	2.08021100
O	-0.64445800	-2.83170400	-1.93106800
O	0.39126900	4.05649500	0.40023100
O	-0.38360000	-3.97826600	-0.01966100
O	2.15904100	-1.05725400	2.55789300
O	-1.79937400	2.40206300	-1.14186500
O	6.64747500	-0.64200200	-1.28136500
O	-6.88218600	-0.91391600	-0.23293200
C	6.18193100	0.24474500	-0.38530400
C	-6.17920500	0.04424600	0.39329400
C	4.69285600	0.20586300	-0.29217900
C	-4.70759900	-0.07453800	0.21400200
C	3.91920600	-0.74358100	-1.17015200
C	-3.87989800	0.96193000	0.92957900
C	2.40567000	-0.52768300	-1.08378300

C	-2.39261900	0.61398800	0.92753200
C	1.92781100	-0.20868500	0.33636300
C	-1.92811700	0.13104700	-0.45441000
C	2.63631600	1.09531200	0.84267500
C	-2.69984700	-1.17342000	-0.82506900
C	4.11661500	1.02312600	0.59758700
C	-4.17345900	-1.02388000	-0.56358900
C	0.42537600	-0.03984500	0.45327500
C	-0.43212800	-0.08754000	-0.56082300
C	2.27268800	-1.38198500	1.26527100
C	-2.23469300	1.19157000	-1.52363600
C	2.37608000	-2.11773200	3.49196100
C	-1.93317000	3.44542200	-2.10830800
C	2.04420800	2.36003300	0.23537800
C	-2.13874500	-2.39222000	-0.11060400
C	2.48805200	2.92593200	-0.89016000
C	-2.62459700	-2.87588200	1.03591800
C	0.92039800	2.98383700	1.00322200
C	-0.98780800	-3.07055300	-0.79071600
C	-0.70937400	4.66331200	1.08017400
C	0.72621200	-4.66298700	-0.61172100
C	8.06704100	-0.68499700	-1.43226200
C	-8.30095400	-0.82763500	-0.09985700
H	4.24640400	-0.62863600	-2.20842000
H	-4.24219500	1.07210000	1.95603900
H	4.16826000	-1.77151800	-0.88840600
H	-4.04709500	1.93935500	0.45791400
H	1.88926800	-1.41349800	-1.46273100
H	-1.80277300	1.47441400	1.25061600
H	2.11513600	0.31140200	-1.72508600
H	-2.20147100	-0.19100700	1.64595700
H	2.46770300	1.16230700	1.92091500
H	-2.56536800	-1.33953500	-1.89853600
H	4.75003700	1.69080600	1.17619600
H	-4.82336800	-1.74808400	-1.04509500
H	0.07825900	0.17494700	1.45963800
H	-0.08509800	-0.31324900	-1.56603200
H	1.65659600	-2.92222600	3.32377500
H	-1.43892400	4.31136900	-1.67104800
H	2.23374800	-1.67246100	4.47519400
H	-1.44797900	3.16138800	-3.04441900
H	3.38933000	-2.51203900	3.39009800
H	-2.98820500	3.65470700	-2.29975500
H	3.32794300	2.50365800	-1.43285100
H	-3.48139700	-2.41450100	1.51711800
H	2.02906000	3.82497700	-1.28382000
H	-2.18307600	-3.74533300	1.50831200
H	-0.93909600	5.56688400	0.51631900
H	1.53425700	-3.95766400	-0.81112500
H	-0.43878000	4.91175300	2.10799000
H	1.04491300	-5.39073800	0.13253200
H	-1.56608800	3.98459800	1.08056400
H	0.41741900	-5.15835400	-1.53460900
H	8.25772000	-1.45040200	-2.18310100
H	-8.59248500	-0.89634900	0.95070800
H	8.44525500	0.28430100	-1.76482200
H	-8.69813100	-1.67029600	-0.66404600
H	8.54339200	-0.94714000	-0.48492100
H	-8.66702100	0.11609600	-0.51048600

2-*RRSS* (X-ray)

0 1

O	6.84395700	-1.38712300	-0.22359800
O	-6.84411900	1.38604900	0.22591100
O	2.71782100	0.81535600	-2.67612700
O	-2.71416500	-0.81499700	2.67718100
O	0.36731300	-2.74974600	-1.97613000
O	-0.36673300	2.75025500	1.97501500
O	0.22328000	-4.15174700	-0.23397800
O	-0.22392700	4.15216600	0.23269600
O	1.90100500	2.31789300	-1.22696400
O	-1.90267400	-2.31819200	1.22575200
O	6.70172500	0.56513100	0.87902500
O	-6.70173800	-0.56546500	-0.87799900
C	6.17006700	-0.48180000	0.22089100
C	-6.17014800	0.48114700	-0.21931100
C	4.68796300	-0.37150700	0.11841700
C	-4.68794400	0.37130800	-0.11762700
C	3.97169900	0.76060800	0.81069900
C	-3.97168600	-0.76022500	-0.81087500
C	2.46252300	0.52940500	0.86131700
C	-2.46250800	-0.52896800	-0.86156400
C	1.91143800	0.05632200	-0.49166600
C	-1.91128700	-0.05618200	0.49142100
C	2.57104000	-1.30752700	-0.87241600
C	-2.57085500	1.30755800	0.87256000
C	4.05278700	-1.28736900	-0.62160500
C	-4.05272400	1.28704800	0.62251600
C	0.40168600	-0.06299300	-0.52521700
C	-0.40151400	0.06323000	0.52490700
C	2.25019300	1.07432900	-1.59195000
C	-2.24940000	-1.07427500	1.59184000
C	2.06713100	3.32596700	-2.22578400
C	-2.06791000	-3.32620600	2.22478700
C	1.92312300	-2.49318500	-0.17284000
C	-1.92353900	2.49333200	0.17261600
C	2.34291100	-2.99012400	0.99389000
C	-2.34409600	2.99020400	-0.99386300
C	0.76839100	-3.11852800	-0.89128100
C	-0.76850200	3.11889900	0.89038100
C	-0.90178000	-4.75709600	-0.87281400
C	0.90147500	4.75765000	0.87081700
C	8.12315200	0.55169300	1.01711200
C	-8.12321500	-0.55231300	-1.01561600
H	4.36139700	0.88246400	1.82556900
H	-4.36154200	-0.88129400	-1.82578400
H	4.19764600	1.70322000	0.29456200
H	-4.19751200	-1.70324000	-0.29545300
H	1.95185300	1.44023000	1.18241700
H	-1.95190800	-1.43972200	-1.18296300
H	2.23557000	-0.24354900	1.60450200
H	-2.23561600	0.24417100	-1.60457700
H	2.42030400	-1.44832500	-1.94720400
H	-2.41951300	1.44836700	1.94726000
H	4.63882200	-2.07991900	-1.07991500
H	-4.63881900	2.07917000	1.08148900
H	-0.00495600	-0.30333100	-1.50385700
H	0.00509800	0.30359900	1.50355000
H	3.12646400	3.46702800	-2.45278900
H	-3.12669000	-3.46380500	2.45637900
H	1.63735600	4.23111500	-1.80046000
H	-1.64302300	-4.23260200	1.79722400
H	1.53902300	3.04632600	-3.13961900
H	-1.53481200	-3.04878800	3.13641200
H	3.20109600	-2.56401000	1.50421400
H	-3.20249300	2.56393100	-1.50369700
H	1.84654400	-3.83611300	1.45422600
H	-1.84817900	3.83629800	-1.45449100
H	-0.65004700	-5.06205300	-1.89026300
H	0.64997100	5.06366400	1.88800900
H	-1.73817600	-4.05365800	-0.89885500
H	1.73761200	4.05394400	0.89742300
H	-1.15116800	-5.62618200	-0.26465300
H	1.15112100	5.62606800	0.26181300
H	8.60178400	0.55128800	0.03525900
H	-8.60152800	-0.55271200	-0.03360800
H	8.37234600	1.45890600	1.56581100
H	-8.37234400	-1.45921300	-1.56486100
H	8.44489700	-0.33367600	1.57009400
H	-8.44537500	0.33335200	-1.56788200

2-*RRRR*

0 1

O	6.64062200	0.00752900	-1.50123400
O	2.89931700	2.47974300	1.88674200
O	0.52842200	-0.88185100	3.10386700

O	0.00973600	-2.80446700	2.07679100
O	2.33861700	3.20109200	-0.16076700
O	6.66699300	-0.98652700	0.51284700
C	6.05807700	-0.33591100	-0.49252500
C	4.61202900	-0.07942000	-0.25425700
C	3.89157900	0.65631100	-1.35303300
C	2.37484800	0.63843100	-1.17241400
C	1.95788200	0.89185700	0.28258600
C	2.56731100	-0.21295400	1.20174100
C	4.01480100	-0.46168900	0.88062300
C	0.45482200	0.93738500	0.48481500
C	2.47184500	2.25481500	0.77777600
C	2.71788500	4.52251400	0.22977400
C	1.78490900	-1.51862000	1.16418300
C	2.02441900	-2.50489400	0.29600800
C	0.72560000	-1.67765500	2.20910700
C	-1.01667800	-3.00147000	3.04987800
C	8.05968700	-1.24260900	0.33061600
C	-0.45486300	0.93743900	-0.48477900
O	-6.64059700	0.00733500	1.50135800
O	-2.89938700	2.47991900	-1.88658000
O	-0.52842000	-0.88128200	-3.10393800
O	-0.00965700	-2.80405900	-2.07720300
O	-2.33871600	3.20113600	0.16098400
O	-6.66697900	-0.98662600	-0.51277000
C	-6.05806700	-0.33602300	0.49261300
C	-4.61203300	-0.07946400	0.25432200
C	-3.89158700	0.65623700	1.35311900
C	-2.37485900	0.63842300	1.17246200
C	-1.95791900	0.89193800	-0.28253300
C	-2.56735300	-0.21281100	-1.20175100
C	-4.01482100	-0.46163700	-0.88059700
C	-2.47186200	2.25493300	-0.77764600
C	-2.71805100	4.52257000	-0.22945000
C	-1.78486400	-1.51842500	-1.16435300
C	-2.02423800	-2.50478800	-0.29624300
C	-0.72554400	-1.67723600	-2.20929900
C	1.01675000	-3.00085100	-3.05034000

C	-8.05965600	-1.24277900	-0.33051200
H	4.25821000	1.68969900	-1.39019200
H	4.16172500	0.22167700	-2.32032300
H	1.96894900	-0.33699200	-1.46394600
H	1.92481100	1.37861400	-1.83608400
H	2.51370200	0.16296600	2.22813300
H	4.58767100	-0.99778600	1.63117900
H	0.15143700	0.94604000	1.52825700
H	2.10675400	4.85990500	1.06987800
H	2.54562700	5.14590000	-0.64624700
H	3.77074000	4.54741500	0.51938600
H	2.82179800	-2.41780100	-0.43585400
H	1.43522400	-3.41332700	0.29746100
H	-1.49456000	-3.94314500	2.78278200
H	-1.73852200	-2.18133900	3.01574600
H	-0.58950500	-3.05739900	4.05329800
H	8.37935000	-1.76722900	1.22989100
H	8.60919300	-0.30541400	0.21708000
H	8.22347300	-1.86137300	-0.55468500
H	-0.15148400	0.94613000	-1.52822300
H	-4.25825900	1.68960800	1.39034700
H	-4.16169400	0.22153600	2.32039000
H	-1.96892600	-0.33700500	1.46393300
H	-1.92482900	1.37858200	1.83616200
H	-2.51380200	0.16321000	-2.22810800
H	-4.58769400	-0.99770300	-1.63117300
H	-3.77091100	4.54744300	-0.51905000
H	-2.10694500	4.86005700	-1.06953300
H	-2.54581800	5.14589500	0.64661900
H	-2.82155200	-2.41780800	0.43570300
H	-1.43493500	-3.41315100	-0.29777600
H	1.49471300	-3.94252300	-2.78338100
H	1.73852900	-2.18066700	-3.01611700
H	0.58955600	-3.05668200	-4.05375600
H	-8.37932000	-1.76737500	-1.22980100
H	-8.60920200	-0.30561400	-0.21692000
H	-8.22339000	-1.86158900	0.5547670

Crystallographic data for compounds 2, 8 and 9

All the compounds were crystallized by slow evaporation from $CHCl_3$. The data collections were carried out at room temperature with a Bruker AXS Smart APEX 3-circle diffractometer equipped with a normal focus sealed tube and an APEX-II CCD area detector. Graphite-monochromated Mo Kα wavelength (λ = 0.71073 Å) was used throughout in conjunction with a nominal power of 50 kV x 30 mA (1500 W) of the X-ray source. Diffraction data were recorded with the same redundant strategy in ω-scan mode, ensuring the collection of 99-100 % complete spheres of reflections up to a maximum Bragg angle (2ϑ) of 52.7 deg or higher. Scan speed and scan width were fixed for each sample. They ranged from 20 to 60 s/frame and from 0.25 to 0.50 deg/frame, depending on the scattering power of the crystal.

The data were integrated with the SAINT+ software package[12] and reduced with SADABS[13] or TWINABS[14] in case of twinning. Empirical corrections were applied for absorption and primary beam anisotropy. The structures were solved by direct methods using shelxs as embedded in the WinGX package[15] and refined with shelxl[16] as detailed below. In all cases, hydrogen atoms were located by Fourier difference and constrained to idealized positions during the least squares refinement.

CCDC 2286201-2286203 contain the supplementary crystallographic data for this paper. These data can be obtained free of charge from The Cambridge Crystallographic Data Centre via www.ccdc.cam.ac.uk/structures.

Compound 2 (CCDC 2286203)

The sample shows pleochroism (from colorless to iridescent) under polarized light. It was cut from a larger agglomerate using a blade and polished by mechanical ablation in a drop of perfluorinated oil.

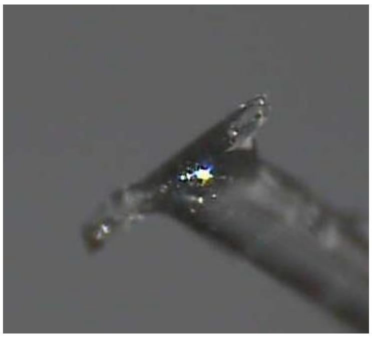

Figure S8. Crystal sample of compound **2** employed for the X-ray experiment. Mounting: on a glass fiber, with bi-component epoxy glue. Specs: prism, transparent, colorless with dimensions 0.575 x 0.100 x 0.075 mm.

The lattice is monoclinic, space group C2/c, nº 15, centric, as unequivocally determined by the analysis of systematic extinctions. Unit cell (Å, deg, Å^3): *a* = 21.9460(8), *b* = 10.7967(4), *c* = 12.6091(5), β = 91.025(2), V = 2987.2(2) as estimated from 2631 intense reflections among 5.2 e 42.4 deg of 2J (final integration result). The asymmetric unit contains ½ molecule, for a total of 4 molecules in cell (density: 1.309 g/cm^3, linear absorption coefficient (μ): 0.101 mm^{-1}).

The dataset consisted of 22124 measured reflections, corresponding to 3437 symmetry-independent data (2099 with I > 2σ(I)) with R_{int} = 0.0370.

The final least squares agreement factors were R1(F) = 0.0498 for the 2099 observed reflections with $F_o > 4\sigma(F_o)$, in conjunction with a goodness-of-fit of 1.025 and largest Fourier residuals of = +0.31 / –0.26 e/Å^3, both at ~ 1.0 Å from the O1 oxygen.

Figure S9 shows the molecular structure, as determined by this X-ray experiment, as well as the absolute configuration of the chiral centers. The compound is achiral and crystallizes in the acentric space group C2/c as a meso-form, with one half molecule per asymmetric unit. Their configurational descriptors are C5(S), C6(S), and C5'(R), C6'(R), with primed labels indicating symmetry-dependent atoms. The two symmetry-related fragments are connected by the central C8=C8' bond with E configuration, which sits on a crystallographic inversion center. Another double bond (C2=C7) is contained in the 6-membered ring and packs

[12] Bruker, SAINT+, **2012**, Bruker AXS Inc., Madison, Wisconsin, USA.
[13] Sheldrick, G. M. SADABS, **1996**, University of Göttingen, Germany.
[14] Sheldrick, G. M. TWINABS, **1996**, University of Göttingen, Germany.
[15] Farrugia, L. J. J. WinGX and ORTEP for Windows : an update. *Appl. Cryst.* **2012**, *45*, 849-854.
[16] Sheldrick, G. M. SHELXT – Integrated space-group and crystal-structure determination. *Acta Cryst.* **2015**, *A71*, 3-8

with a Z configuration with respect to the conjugated carbonyl O1. The last double bond is C11=C12, with the terminal CH_2 sitting at a dihedral of 180 deg with respect to the conjugated O3 carbonyl.

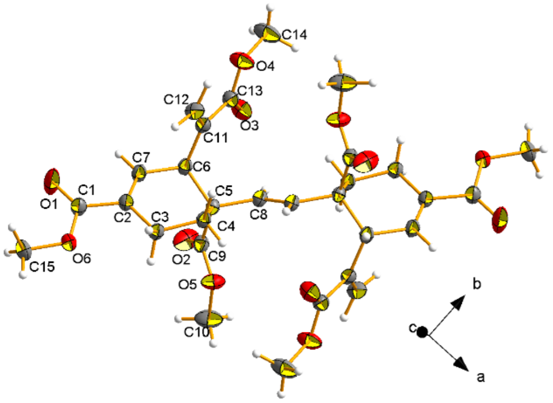

Figure S9 Molecular structure of **2** at RT, with the atom-numbering scheme. Thermal ellipsoids of non-H atoms were drawn at the 30 % probability level. The usual color code was employed for atoms (grey: C; white: H; red: O).

The puckering analysis[17] shows that the cyclohexene ring is not completely planar. Configurational descriptors of the C2–C3–C4–C5–C6–C7 6-membered ring (Q = 0.4975, θ = 52.39 deg, φ = 160.49 deg) are compatible with a distorted half-chair conformation.

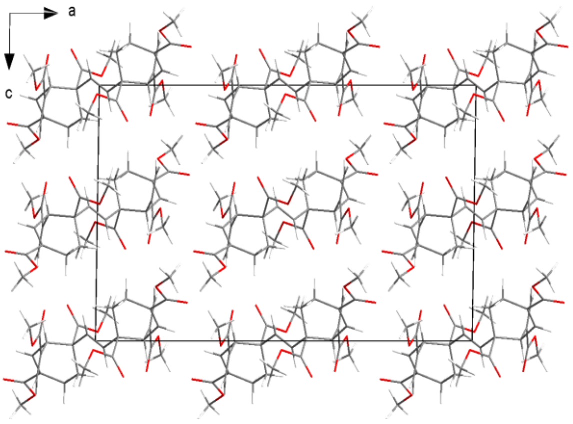

Figure S10. Crystal packing of compound **2** at RT, as seen along the *b* cell axis. Color code as in Figure S9.

Figure S10 shows the main packing motifs of compound **2** along the *b* cell axis. No strong hydrogen bond donors are present, so the only relevant contacts with distance H···acceptor lower than the sum of the van der Waals radii are C–H···O ones that involve methyl carbon and keto oxygens. Overall, the packing is likely dominated by steric requirements, with translation-dependent molecules piled up along the short cell axis *b* (Figure S10), while along *c* the neighboring moieties are arranged so that their central double bond C8=C8' are oriented almost perpendicularly. Terminal ester groups are projected in the free space among the crowded hydrocarbon cores.

[17] Cremer, D; Pople, J.A. General definition of ring puckering coordinates. *J. Am. Chem. Soc.* **1975**, *97*, 1354-1358.

Compound 8 (CCDC 2286201)

The sample shows pleochroism (colorless to grey). It was cut from an agglomerate through a stainless-steel micro-blade and polished by mechanical ablation in a perfluorinated oil drop. The specimen is a ~ 50 % epitaxial twin.

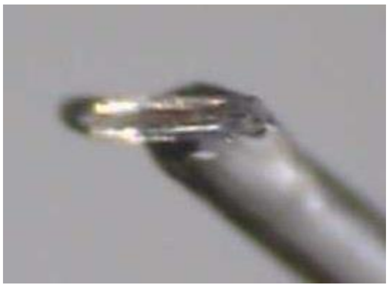

Figure S11. Crystal sample of compound **8** employed for the X-ray experiment. Mounting: on a glass fiber, with a drop of perfluorinated oil. Specs: prism, colorless, transparent, with dimensions ≈ 0.275 x 0.100 x 0.050 mm.

The lattice is triclinic, space group, $P\bar{1}$, nº 2, centric. Unit cell (Å, deg, Å^3): a = 5.7827(17), b = 9.089(3), c = 10.855(3), α = 67.179(15), β = 87.660(17), γ = 84.446(16), V = 523.4(3) as estimated from 888 intense reflections among 4.8 and 43.1 deg in 2ϑ (final integration result).The asymmetric unit contains ½ molecule, for a total of 1 molecule in cell (density: 1.378 g/cm^3, linear absorption coefficient (μ): 0.100 mm^{-1}).

The dataset consisted of a total of 6075 measured reflections and R_{int} = 0.0230 for each lattice. The least squares refinement was carried out on a full HKLF5 file containing the reflections from both twins. The BASF parameter, expressing the contribution of the twin components, refined to 0.50(3) in conjunction with 100 % superimposition of the recorded intensities and also in agreement with the TWINABS estimate.

The final least squares agreement factors were R1(F) = 0.0591 for the 3298 observed reflections with $F_o > 4\sigma(F_o)$, in conjunction with a goodness-of-fit of 1.004 and largest Fourier residuals of +0.39 / –0.2 e/Å^3.

Figure S12 shows the molecular structure, as determined by this X-ray experiment. The compound crystallizes in the centric space group $P\bar{1}$, with ½ formulae in the asymmetric unit and 1 molecule per cell as a meso form (C9 has descriptor *S*). In the crystal, the compound **8** has an intramolecular crystallographic center of symmetry that lies at the midpoint of the central C=C bridge.

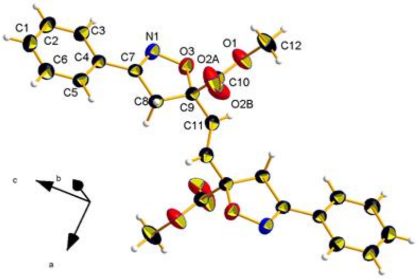

Figure S12. Molecular structure of **8** at RT, with the atom-numbering scheme highlighted for the symmetry–independent part of the molecule. The O2 carbonyl oxygen is disordered over two non–crystallographic positions. Thermal ellipsoids at RT were drawn at the 30 % probability level. Atoms are represented with the following color code (C: black; N: blue; O: red; H: white).

The 5-membred isoxazole–like ring has an essentially perfect envelope conformation, with puckering amplitude[17] of 0.150(3) Å and phase 396(1) deg. The C9 atom lies 0.204 Å apart from the mean least–squares plane computed from the atomic coordinates of atoms C7, C8, N1 and O3 (see Figure S12 for the atom numbering).

The carboxy O2 atom is positionally disordered, with site occupation factors refining to 50(3) % occupancy in the final least–squares model. Being not connected to any conjugated system, the trans C9-C11 and C9a-C11a bonds are rotatable, and the two parts of the molecule can undergo a significant librational motion with respect to each other.

As for the crystal packing, the compound **8** lacks strong hydrogen bond (HB) donors. Thus, no significant hydrogen bonds can be set up. Crystal packing is likely dominated by dispersive–repulsive balance, being odd–order electrostatic moments set to zero by symmetry. This might account for the difficulty of obtaining good diffracting crystals. The molecule assumes an extended conformation, with terminal phenyl rings at the opposite ends of the hydrocarbon chain, roughly along the c direction (Figure S13).

Stacking interactions are set up among benzene rings lying parallel to the (a,b) plane, which form infinite ladders running along the [010] direction (Figure S13).

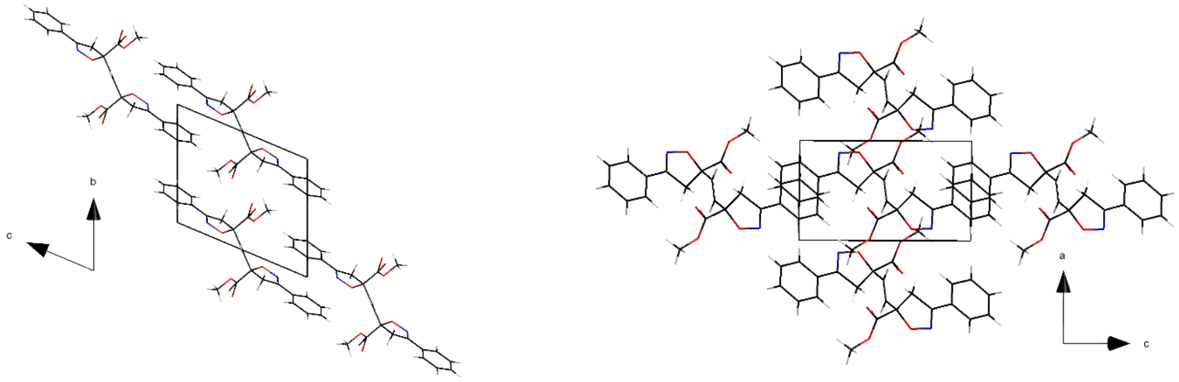

(a) (b)

Figure S13. Wires–stick representation of the crystal packing of **8** at RT, as seen along the *a* (a) and *b* (b) cell axes. Color code as in Figure S12.

Compound 9 (CCDC 2286202)

The sample shows pleochroism (darkening). It was cut from an agglomerate through a stainless-steel micro-blade and mounted at the top of a capillary fiber.

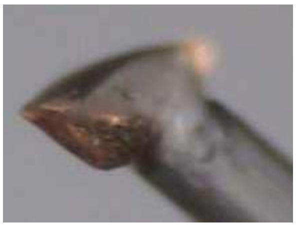

Figure S14. Crystal sample of compound **9** employed for the X-ray experiment. Mounting: on a glass fiber, with bi-component epoxy glue. Specs: prism, pale red, transparent, with dimensions ≈ 0.450 x 0.250 x 0.225 mm.

The lattice is triclinic, space group, P $\bar{1}$, nº 2, centric. Unit cell (Å, deg, Å^3): a = 9.4163(2), b = 9.7420(2), c = 11.5577(2), α = 76.914(1), β =85.107(1), γ= 85.550(1), V = 1027.08(4), as estimated from 2978 intense reflections among 3.0 and 44.6 deg in 2ϑ (final integration result. The asymmetric unit contains ½ molecule, for a total of 1 molecule in cell (density: 1.337 g/cm^3, linear absorption coefficient (μ): 0.100 mm^{-1}).

The dataset consisted of a total of 15614 measured reflections and R_{int} = 0.0225. The final least squares agreement factors were R1(F) = 0.0467 for the 3036 observed reflections with $F_o > 4\sigma(F_o)$, in conjunction with a goodness-of-fit of 1.023 and largest Fourier residuals of +0.20 / –0.17 e/Å^3.

Figure S15 shows the molecular structure, as determined by this X-ray experiment. The compound is achiral and crystallizes in the centric space group P $\bar{1}$ as a meso form, with 1 formula in cell and ½ molecule in the asymmetric unit. The configuration of the chiral centers of one symmetry–independent fragment, *i.e.* C9 (S), C12 (R) and C13 (S), is also shown. The two inversion–dependent half molecules are bridged by a vinyl group (C20=C20') in E configuration.

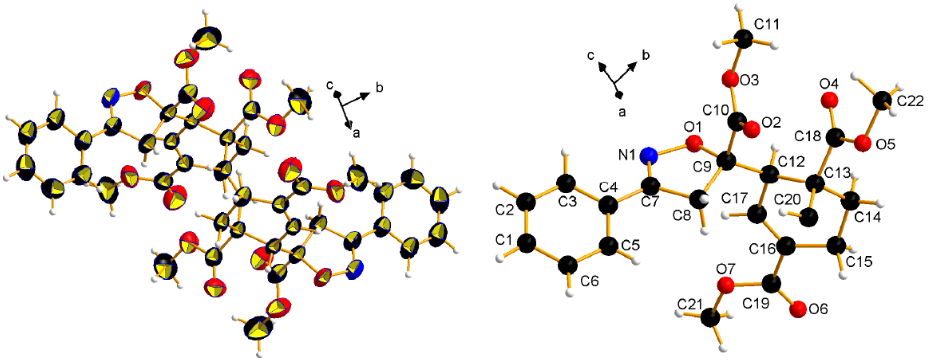

Figure S15. Left: molecule of compound **9**. Thermal ellipsoids at RT were drawn at the 50 % probability level. Atoms are represented with the usual color code (C: black; N: blue; O: red; H: white). Right: asymmetric unit of **9** (1/2 molecule), with the atom numbering scheme. The full structure is generated by an inversion center that lies at the center of the bridging C=C vinyl group at the carbon C20.

The partially saturated isoxazole ring adopts an envelope conformation on C9, with puckering amplitude[17] Q = 0.2130 Å and phase ϕ = 139.95 deg. The atom C9 lies 0.34 Å away from the least–squares plane containing the other atoms of the 5–membered ring. The only other puckered ring is the cyclohexenyl substituent, which adopts a distorted half–chair conformation with puckering parameters Q = 0.5064 Å, θ = 50.78 deg and ϕ = 80.03 deg.

As for the crystal packing, neither significant hydrogen bond donors nor dipole–dipole interactions are present in compound **9**; accordingly, the crystal structure is likely dominated by the tradeoff among van der Waals attractive interactions and short–range repulsions. Molecules are oriented so that the central C=C bond roughly follows the *a* direction (Figure S16), while bulky phenyl groups are arranged along *c*. –COOMe groups extend in the (*a*,*b*) plane and possible CH···π short contacts are set up with facing aromatic rings along the [011] direction. The molecule itself is folded so that the distance among the largest substituents is maximized, with phenyl rings being almost perpendicular to the cyclohexenyl groups: the dihedral angles among the corresponding mean least–square planes are ~74 deg.

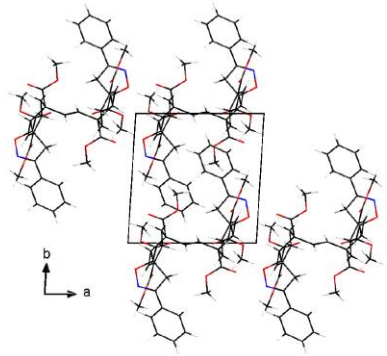

Figure S16. Wires–stick representation of the crystal packing of **9** at RT, as seen along the *c* cell axis. Color code as in Figure 15.

^{1}HNMR and ^{13}CNMR

Benzyl 2-(hydroxymethyl)-acrylate

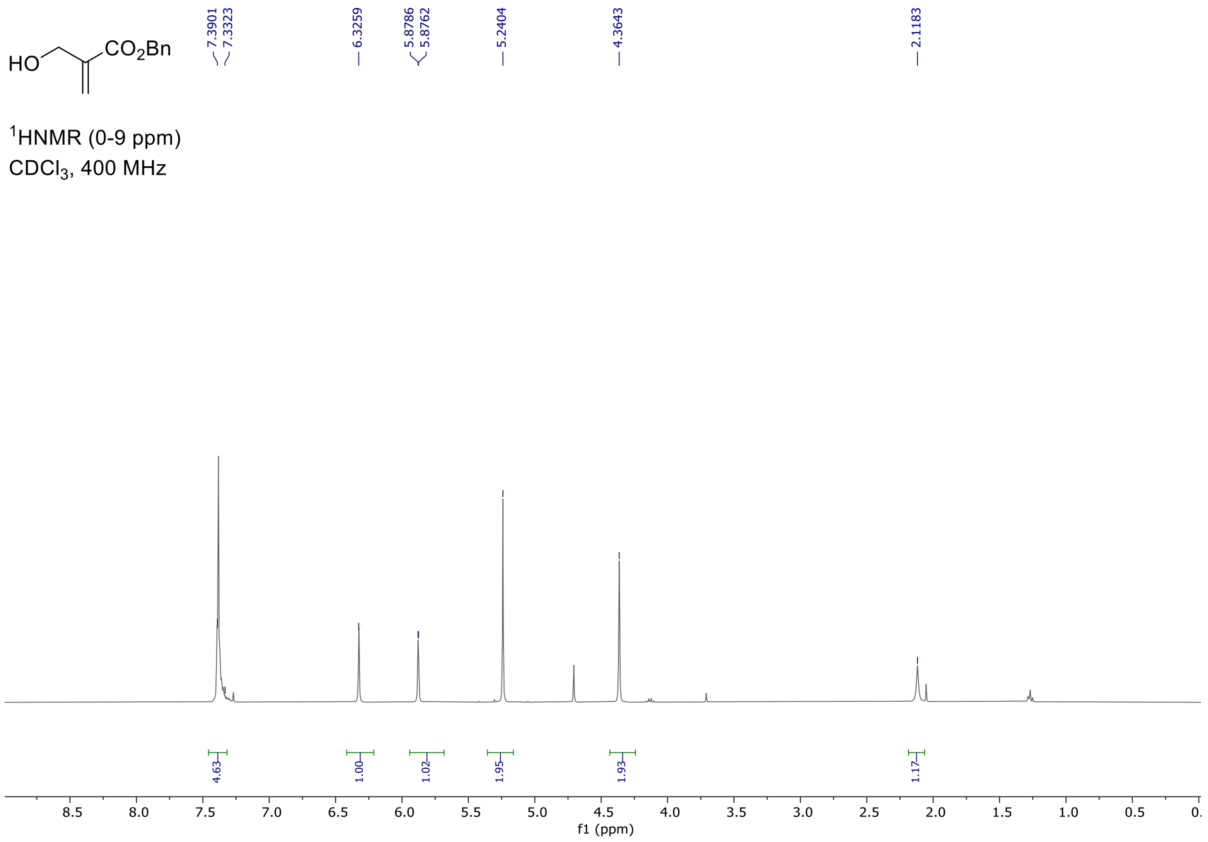

Butyl 2-(hydroxymethyl)-acrylate

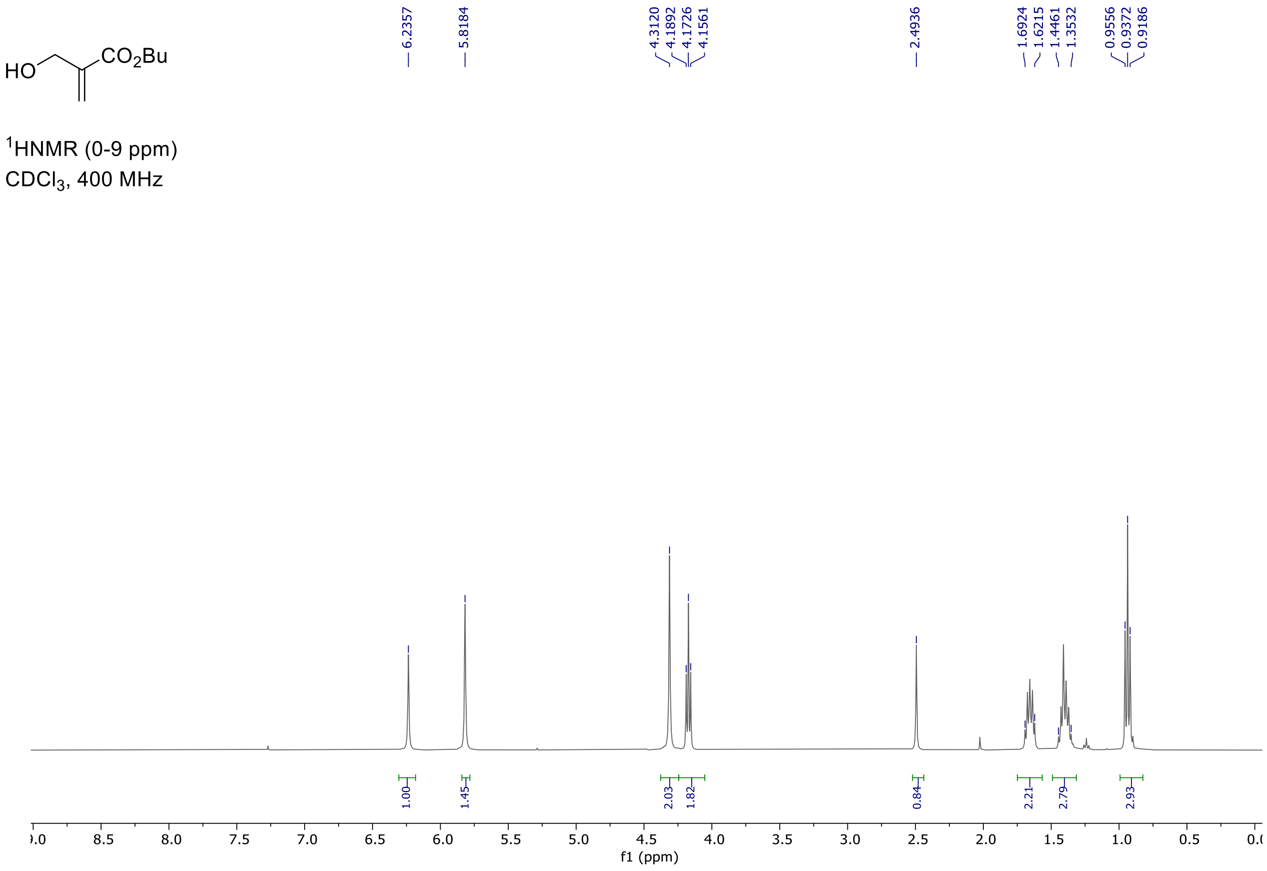

t-Butyl 2-(hydroxymethyl)-acrylate

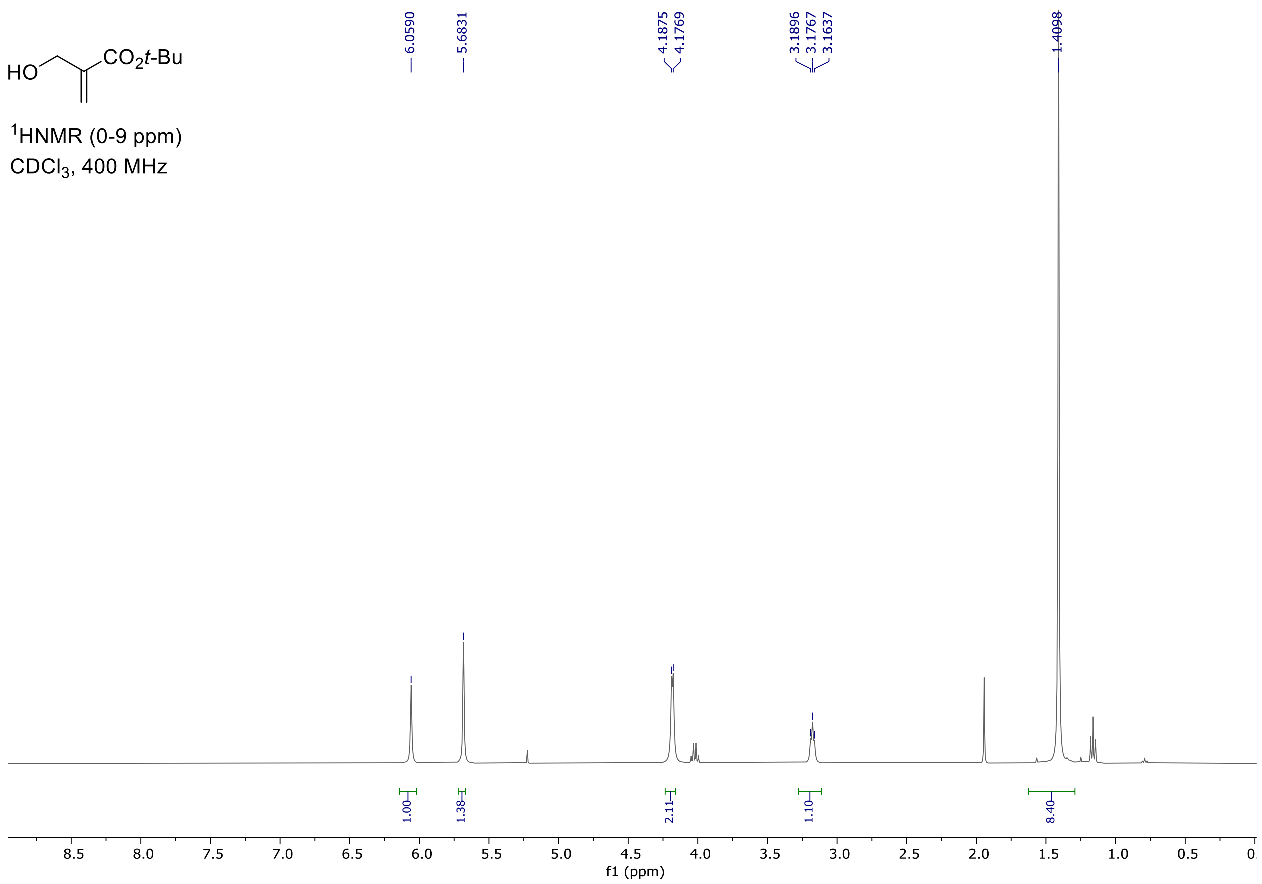

Benzyl 2-(bromomethyl)-acrylate (1f)

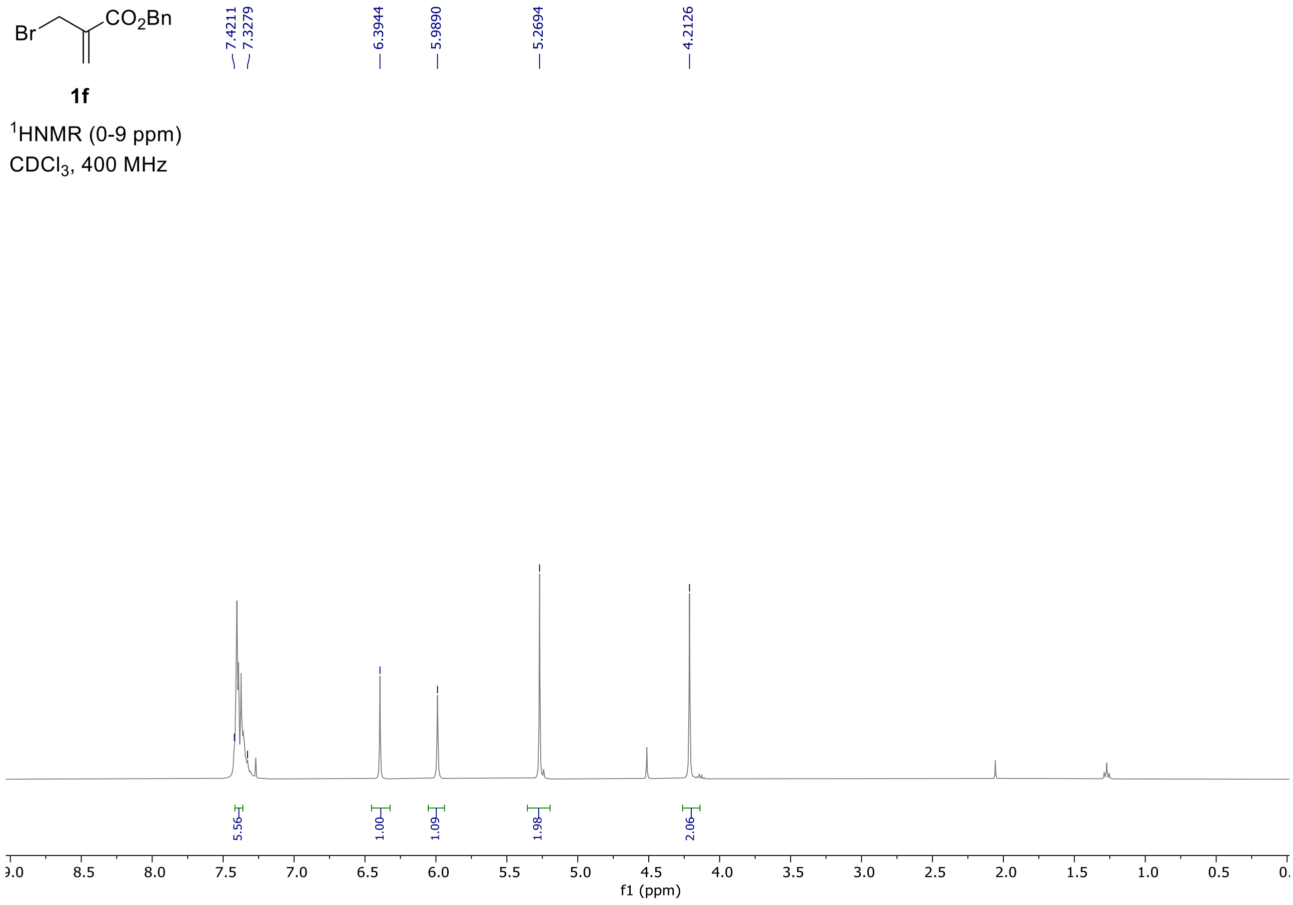

Butyl 2-(bromomethyl)-acrylate (1g)

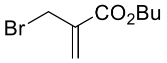

1g

^{1}HNMR (0-9 ppm)

$CDCl_3$, 400 MHz

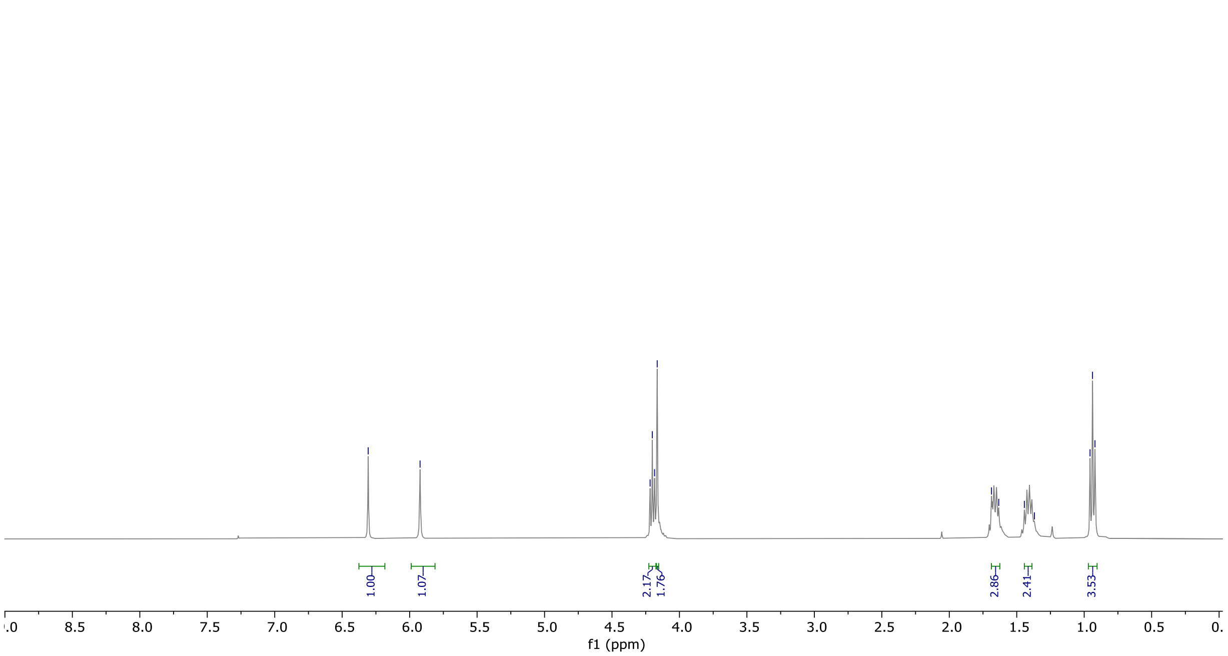

t-Butyl 2-(bromomethyl)-acrylate (1h)

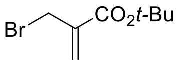

1h

^{1}HNMR (0-9 ppm)

$CDCl_3$, 400 MHz

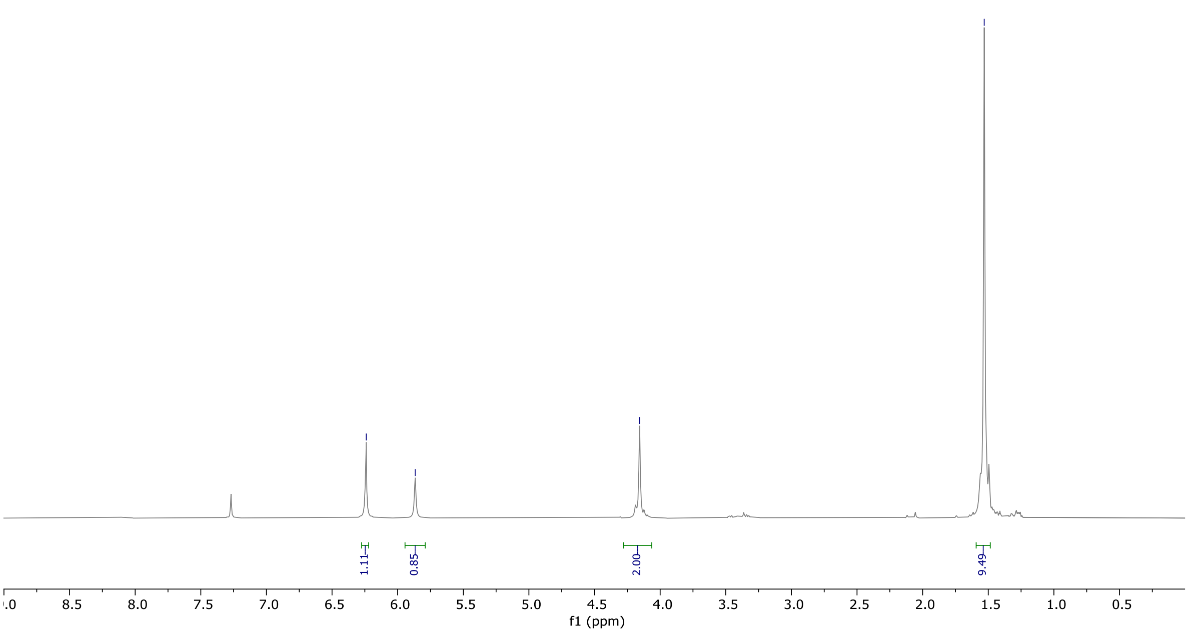

1,2-Bis[2-(1-(methoxycarbonyl)eth-1-en-1yl)-1,4-di(methoxycarbonyl)cyclohex-3-en-1-yl]ethene (2)

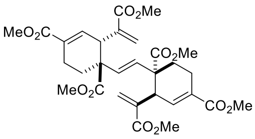

2

^{1}HNMR (0-9 ppm)

$CDCl_3$, 400 MHz

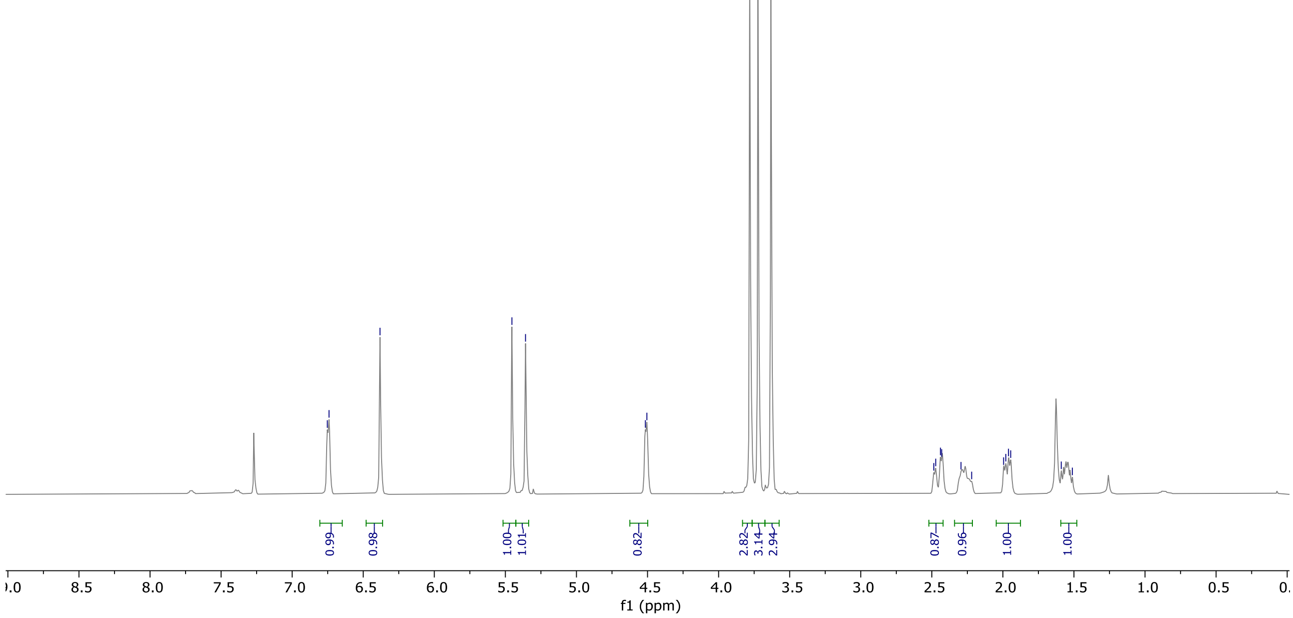

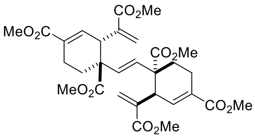

2

^{13}CNMR- APT (0-200 ppm)

$CDCl_3$, 101 MHz

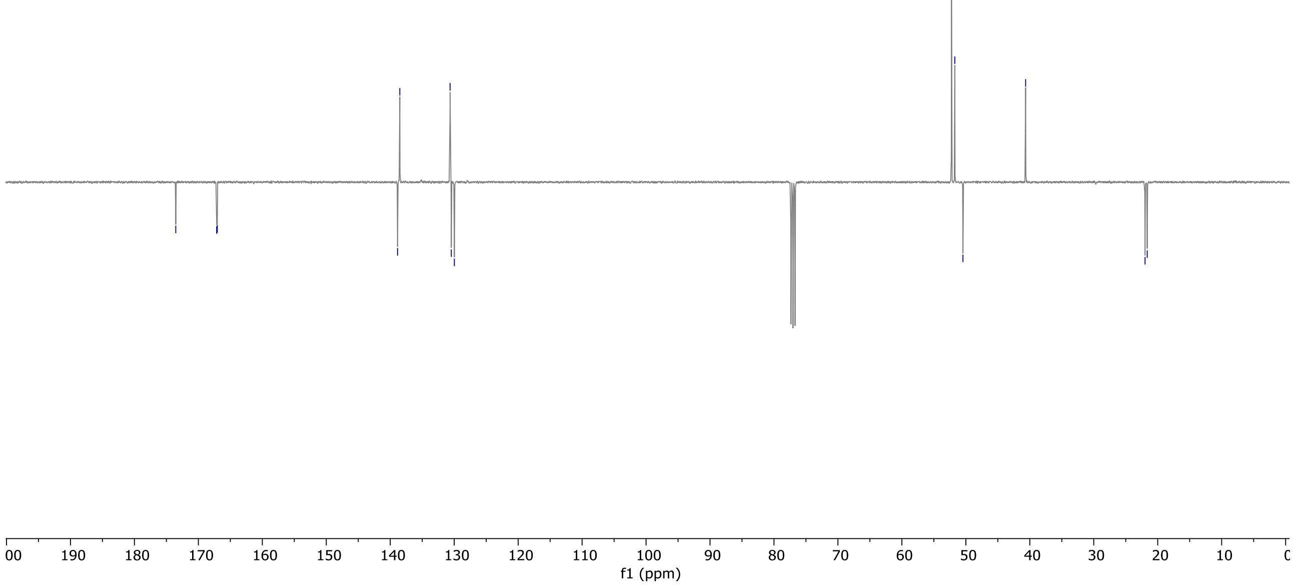

1,2-Bis[2-(1-(ethoxycarbonyl)eth-1-en-1yl)-1,4-di(ethoxycarbonyl)cyclohex-3-en-1-yl]ethene (3)

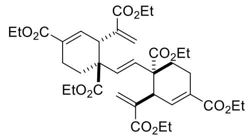

3

^{1}HNMR (0-9 ppm)
$CDCl_3$, 400 MHz

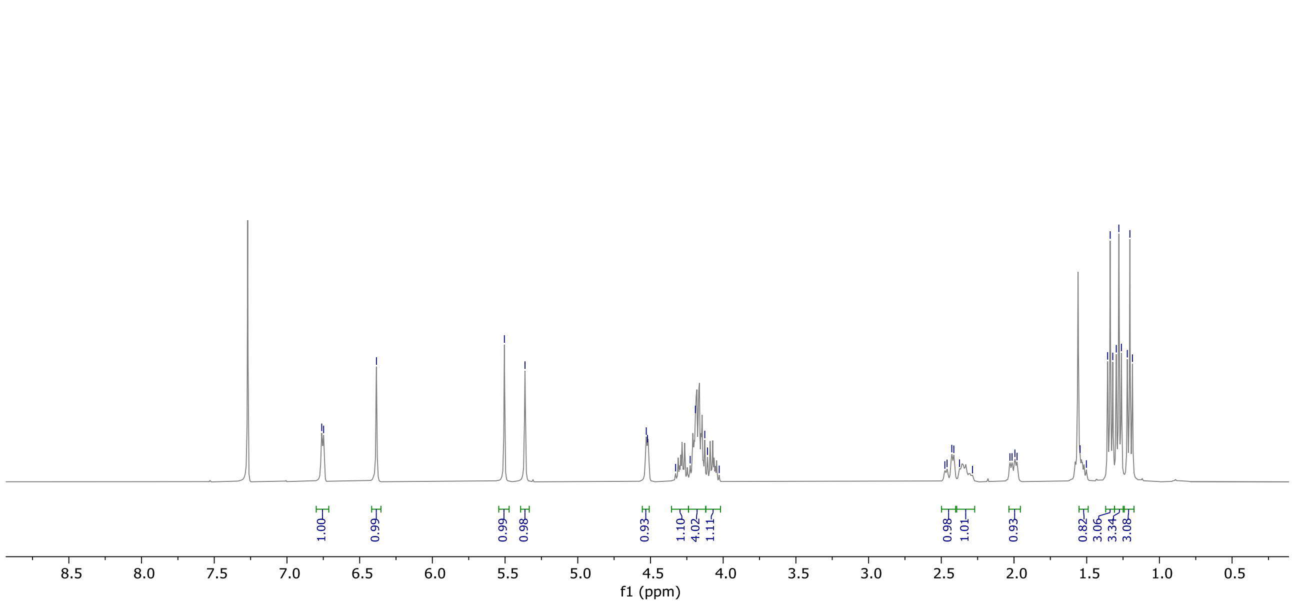

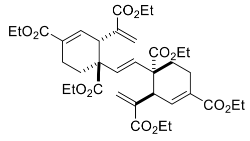

3

^{13}CNMR- APT (0-200 ppm)
$CDCl_3$, 101 MHz

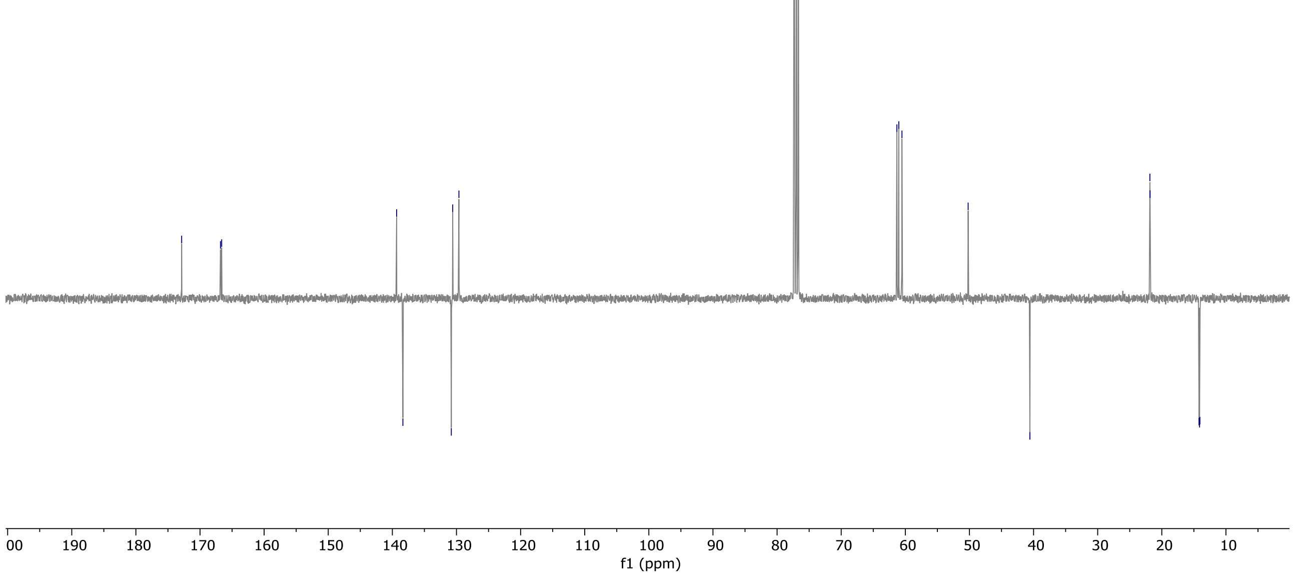

1,2-Bis[2-(1-(benzyloxycarbonyl)eth-1-en-1yl)-1,4-di(benzyloxycarbonyl)cyclohex-3-en-1-yl]ethene (4)

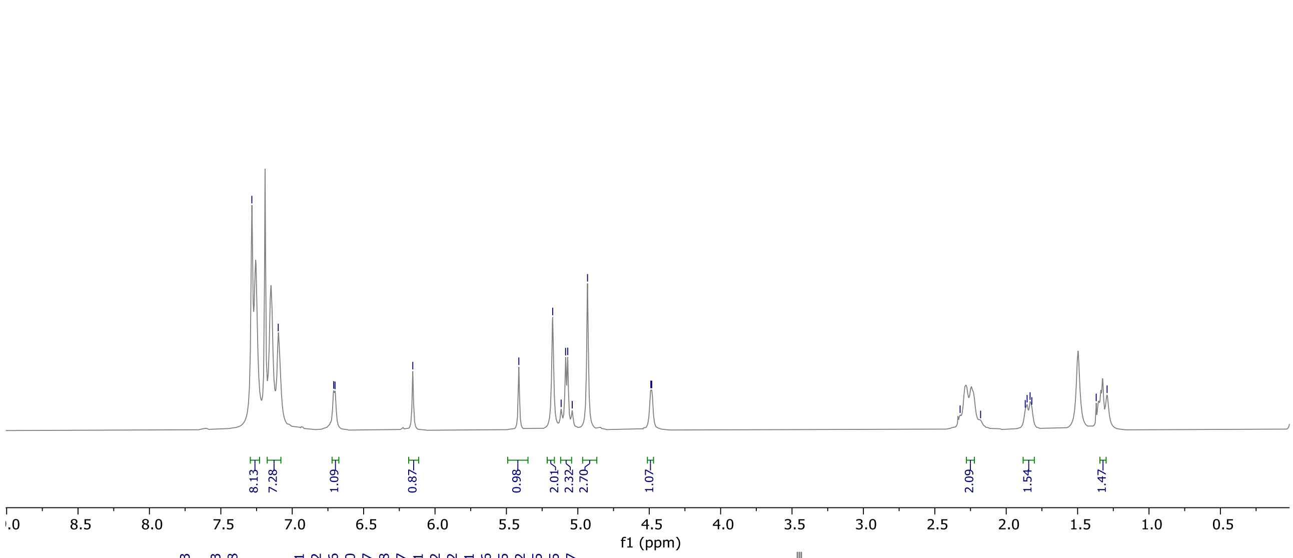

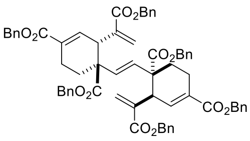

4

^{1}HNMR (0-9 ppm)

$CDCl_3$, 400 MHz

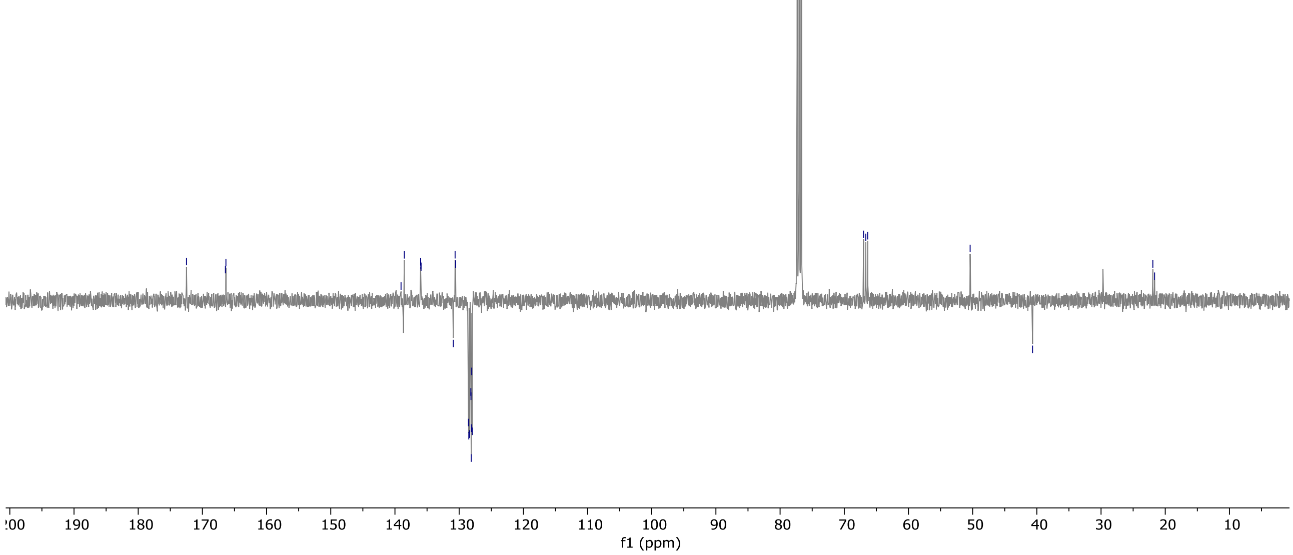

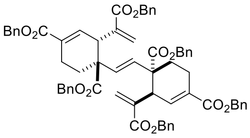

4

^{13}CNMR- APT (0-200 ppm)

$CDCl_3$, 101 MHz

1,2-Bis[2-(1-(butyloxycarbonyl)eth-1-en-1yl)-1,4-di(butyloxycarbonyl)cyclohex-3-en-1-yl]ethene (5)

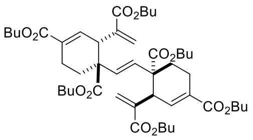

5

^{1}HNMR (0-9 ppm)

$CDCl_3$, 400 MHz

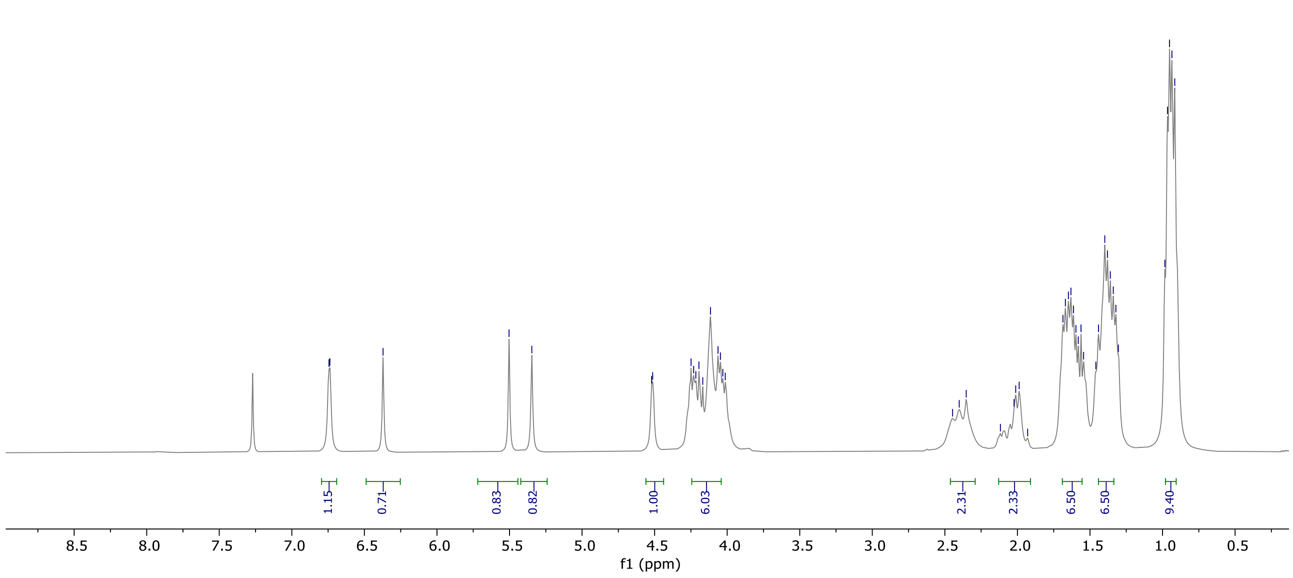

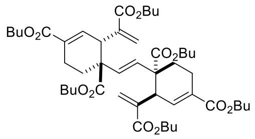

5

^{13}CNMR- APT (0-200 ppm)

$CDCl_3$, 101 MHz

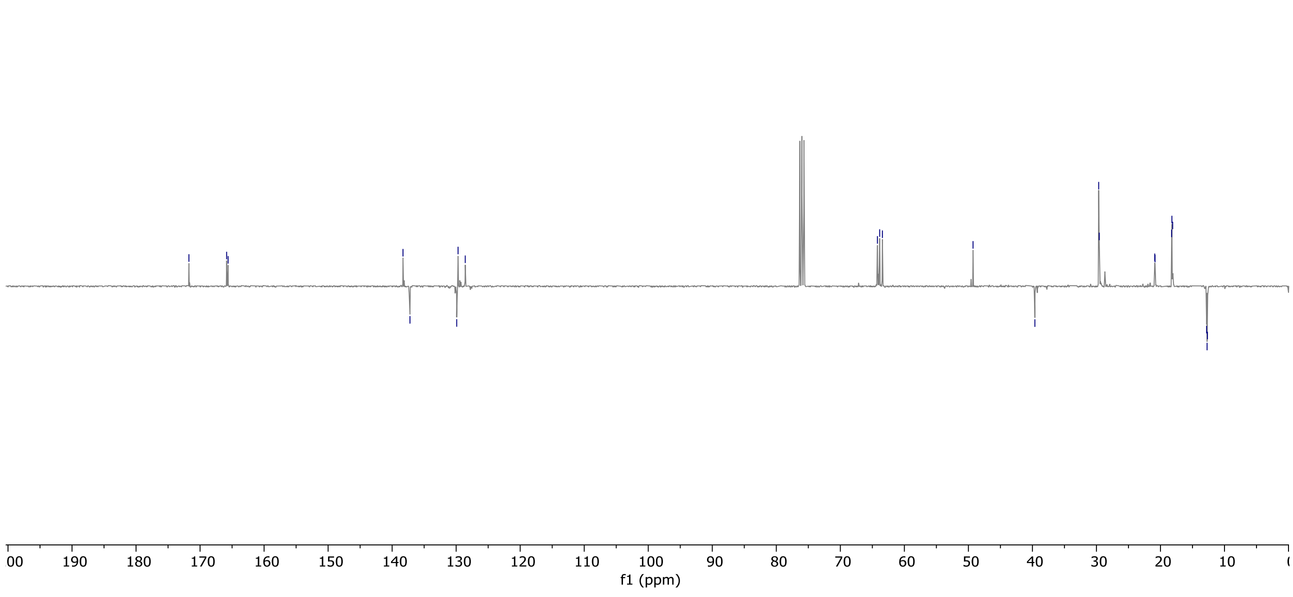

1,2-Bis[2-(1-(*t*-butyloxycarbonyl)eth-1-en-1yl)-1,4-di(*t*-butyloxycarbonyl)cyclohex-3-en-1-yl]ethene (6)

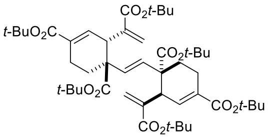

6

^{1}HNMR (0-9 ppm)

$CDCl_3$, 400 MHz

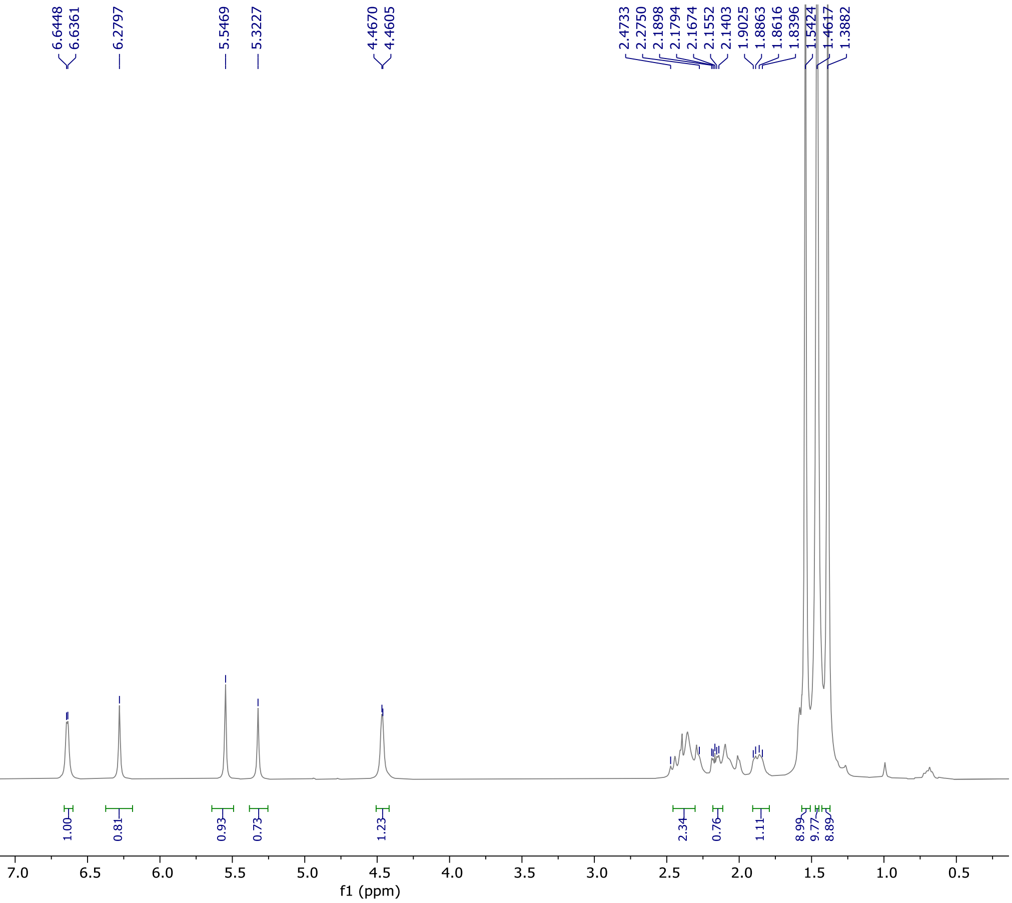

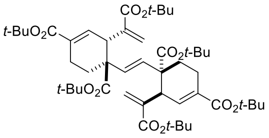

6

^{13}CNMR- APT (0-200 ppm)

$CDCl_3$, 101 MHz

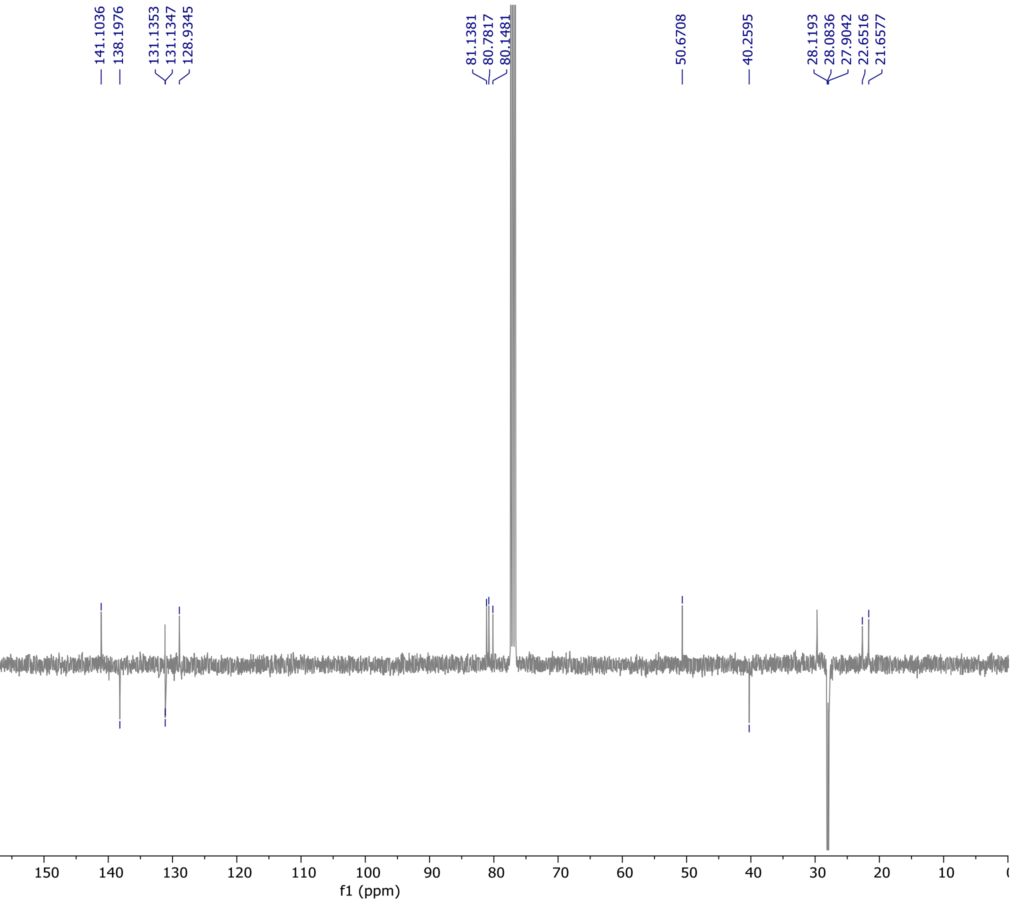

Compound 8

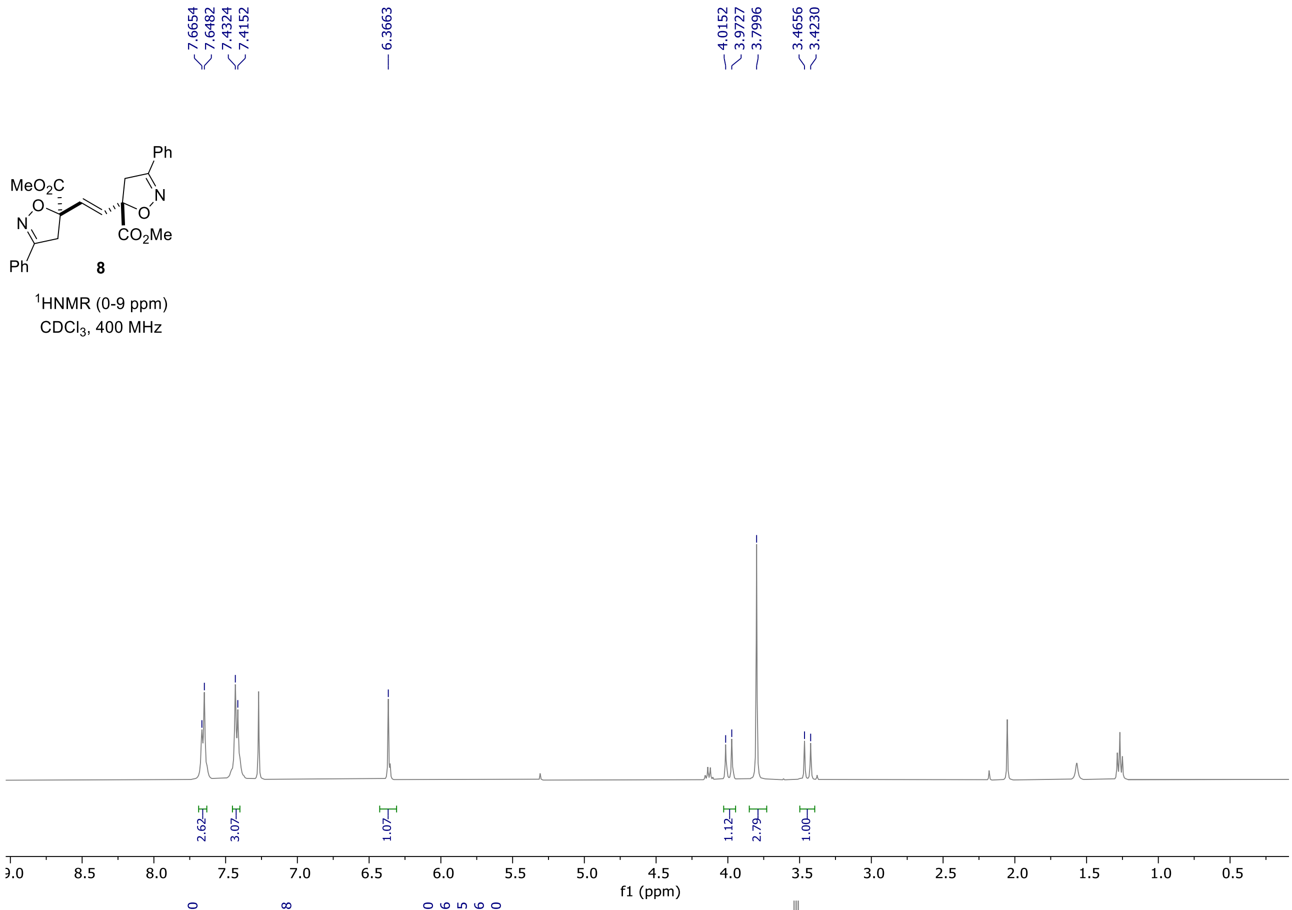
7.6654
7.6482
7.4324
7.4152
6.3663
4.0152
3.9727
3.7996
3.4656
3.4230
MeO2C
Ph
N
O
CO2Me
8
1HNMR (0-9 ppm)
CDCl3, 400 MHz
2.62
3.07
1.07
1.12
2.79
1.00
f1 (ppm)

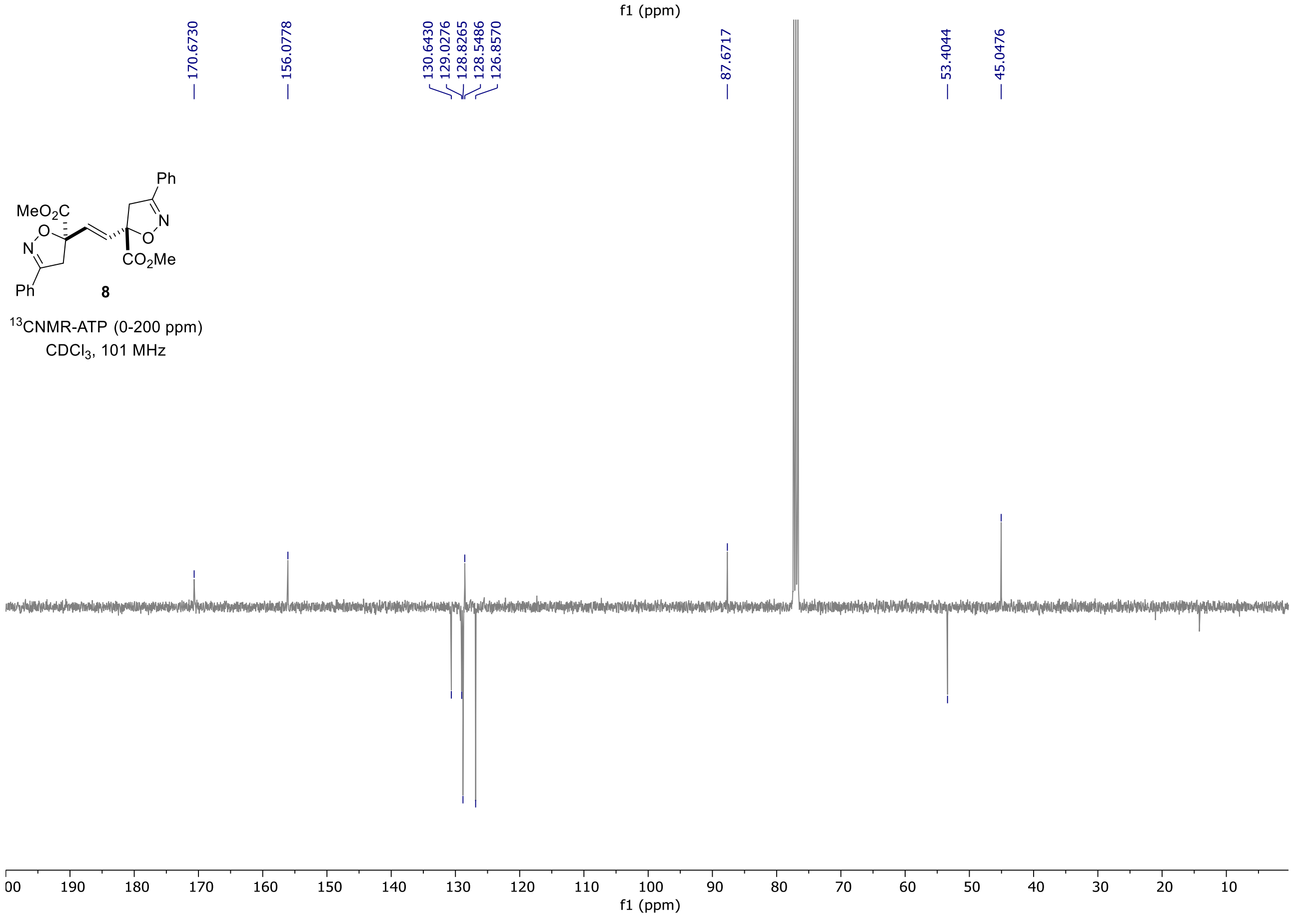
170.6730
156.0778
130.6430
129.0276
128.8265
128.5486
126.8570
87.6717
53.4044
45.0476
MeO2C
Ph
N
O
CO2Me
8
13CNMR-ATP (0-200 ppm)
CDCl3, 101 MHz
f1 (ppm)

Compound 9

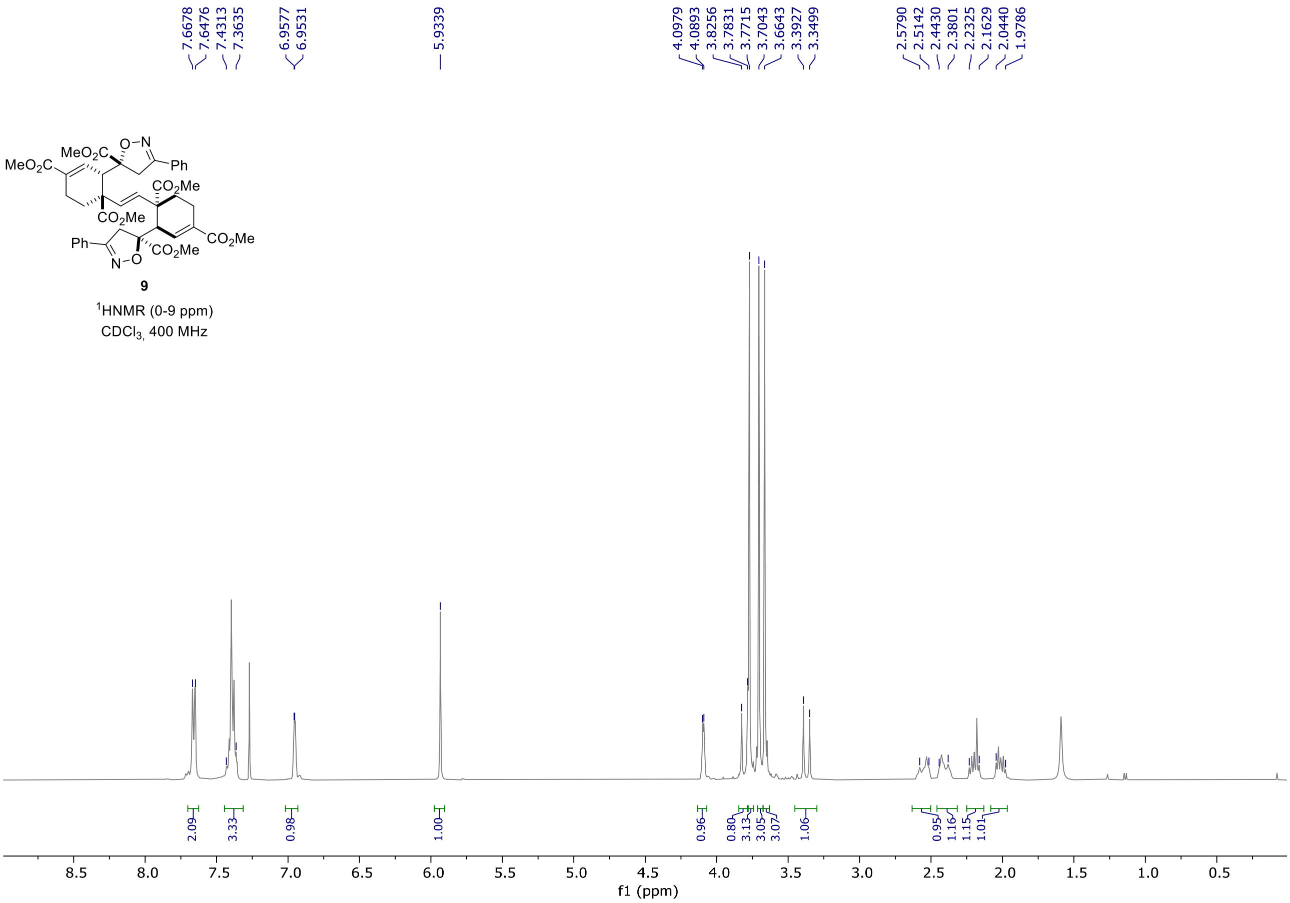

7.6678
7.6476
7.4313
7.3635
6.9577
6.9531
5.9339
4.0979
4.0893
3.8256
3.7831
3.7715
3.7043
3.6643
3.3927
3.3499
2.5790
2.5142
2.4430
2.3801
2.2325
2.1629
2.0440
1.9786
MeO2C
MeO2C
O–N
Ph
CO2Me
CO2Me
Ph
CO2Me
CO2Me
N–O
9
1HNMR (0-9 ppm)
CDCl3, 400 MHz
2.09
3.33
0.98
1.00
0.96
0.80
3.13
3.05
3.07
1.06
0.95
1.16
1.15
1.01
8.5
8.0
7.5
7.0
6.5
6.0
5.5
5.0
4.5
4.0
3.5
3.0
2.5
2.0
1.5
1.0
0.5
f1 (ppm)

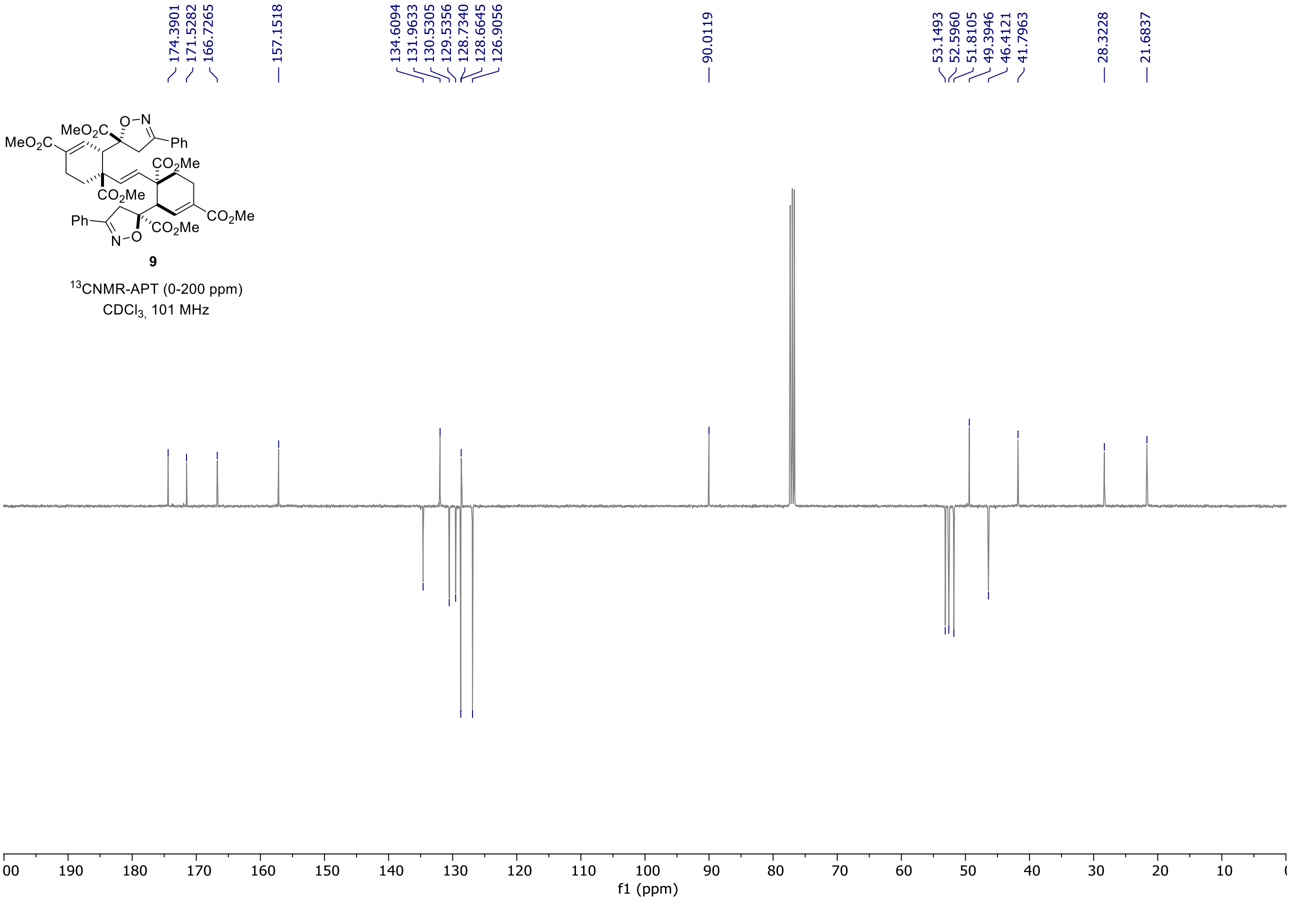

174.3901
171.5282
166.7265
157.1518
134.6094
131.9633
130.5305
129.5356
128.7340
128.6645
126.9056
90.0119
53.1493
52.5960
51.8105
49.3946
46.4121
41.7963
28.3228
21.6837
MeO2C
MeO2C
O–N
Ph
CO2Me
CO2Me
Ph
CO2Me
CO2Me
N–O
9
13CNMR-APT (0-200 ppm)
CDCl3, 101 MHz
190
180
170
160
150
140
130
120
110
100
90
80
70
60
50
40
30
20
10
f1 (ppm)
